# Trifluridine/tipiracil enhances radiation-induced abscopal effects and augments PD-1 blockade in gastric cancer

**DOI:** 10.1038/s41598-026-53689-9

**Published:** 2026-05-21

**Authors:** Tetsuya Asakawa, Jun Kinoshita, Kenta Doden, Kengo Hayashi, Saki Hayashi, Hiroto Saito, Toshikatsu Tsuji, Daisuke Yamamoto, Hideki Moriyama, Sachiyo Nomura, Yasuhiko Yamamoto, Noriyuki Inaki

**Affiliations:** 1 Department of Gastrointestinal Surgery, Graduate School of Medical Science, Kanazawa University, 13-1 Takara-machi, Kanazawa, 920-8641 Ishikawa Japan; 2 Clinical Pharmaceutical Sciences, School of Pharmacy and Pharmaceutical Sciences, Hoshi University, Shinagawa-ku, Tokyo, 142-8501 Japan; 3 Isotope Science Center, The University of Tokyo, Bunkyo-ku, Tokyo, 113-0033 Japan; 4 Department of Gastrointestinal Surgery, The University of Tokyo Hospital, Bunkyo-ku, Tokyo, 113-8655 Japan; 5 Department of Biochemistry and Molecular Vascular Biology, Kanazawa University Graduate School of Medical Sciences, Kanazawa, 920-8640 Ishikawa Japan

**Keywords:** Gastric cancer, Trifluridine/tipiracil, Immunogenic cell death, Abscopal effect, Immune checkpoint inhibitors, Cancer, Immunology, Oncology

## Abstract

Gastric cancer (GC) often exhibits resistance to anti-programmed death-1 (PD-1) immunotherapy. Immunogenic cell death (ICD) enhances antitumor immunity, and trifluridine/tipiracil (FTD/TPI) induces ICD and modulates immunity. Radiation therapy (RT) may trigger abscopal effects. We investigated whether FTD/TPI combined with RT enhances tumor immunity in GC and its impact with PD-1 blockade. ICD induction by FTD and RT in YTN16 mouse GC cells was assessed by calreticulin, high-mobility group box 1 (HMGB1), and ATP evaluation. A dual subcutaneous YTN16 tumor model was established in C57BL/6J mice; mice were treated with FTD/TPI and RT (with irradiation of the first tumor). ICD was induced in vitro by FTD or RT and enhanced by the combination, as indicated by increased calreticulin surface expression and HMGB1 and ATP release. ICD induction in the first tumor was confirmed by HMGB1 release. FTD/TPI + RT suppressed growth of the second tumor and enhanced antitumor immunity by increasing CD8⁺ T-cell infiltration and depleting M2 macrophages. PD-1 expression on CD8⁺ T cells increased after FTD/TPI + RT; adding anti-PD-1 further suppressed the second tumor growth. Our findings support clinical trials of this triple-combination strategy in advanced GC, particularly in subgroups refractory to immune checkpoint blockade.

**Supplementary Information:**

The online version contains supplementary material available at 10.1038/s41598-026-53689-9.

## Introduction

Gastric cancer (GC) is the fourth leading cause of cancer-related death and remains a therapeutic challenge despite advances in systemic therapies^1,2^. While immune checkpoint inhibitors (ICIs) have shown promising results in various malignancies, their efficacy in GC is limited, with response rates of ~ 11% in third- or later-line settings^3^. Even in first-line treatment, the benefit of ICI-based regimens is largely confined to patients with high programmed death-ligand 1 (PD-L1) expression^4^. More effective therapeutic strategies are therefore needed for immunotherapy-refractory populations^5,6^.

One approach to overcome resistance to immunotherapy is the induction of immunogenic cell death (ICD), which activates antitumor immunity by promoting dendritic cell activation and T-cell priming through the release of damage-associated molecular patterns (DAMPs) such as calreticulin (CRT), ATP, and high-mobility group box 1 (HMGB1)^7–10^. Radiation therapy (RT) is a potent inducer of ICD and has been shown to elicit immune-mediated regression of non-irradiated tumors, known as the abscopal effect. However, this phenomenon is rarely observed clinically^11–13^, a finding that is generally attributed to the inherently paradoxical immunological effects of RT. While RT promotes antitumor immune activation, it concurrently drives immunosuppressive remodeling of the tumor microenvironment, characterized by the accumulation of M2 macrophages and regulatory T cells^14–17^. Thus, rational combination strategies that enhance the immunogenic effects of RT are required to achieve robust systemic antitumor immunity.

Trifluridine/tipiracil (FTD/TPI) is an oral chemotherapeutic agent approved for the treatment of metastatic colorectal cancer and advanced GC^18,19^. FTD is a nucleoside analogue whose principal mechanism of action is direct incorporation into DNA, distinguishing it from conventional antimetabolites such as 5-fluorouracil^20–22^. This DNA-directed cytotoxicity has been shown to induce ICD, yet in vivo evidence for the immunological impact of FTD/TPI remains limited. Only one preclinical study has demonstrated that FTD/TPI—particularly in combination with oxaliplatin—induced ICD and modulated the tumor immune microenvironment in a murine colorectal cancer model^23^. The immunological and therapeutic potential of combining FTD/TPI with RT has not been explored.

In this study, we investigated the immunogenic and systemic effects of FTD/TPI combined with RT in a syngeneic GC model. We evaluated whether FTD/TPI amplifies RT-induced ICD and reshapes the tumor immune microenvironment, particularly macrophage polarization and dendritic cell activation, to promote systemic CD8⁺ T-cell-mediated antitumor immunity. Furthermore, we examined whether this combination therapy potentiates the efficacy of anti-programmed death-1 (PD-1) immunotherapy in vivo. Our findings establish a novel radio-chemoimmunotherapy platform and provide a rationale for future clinical translation, particularly for patients with GC refractory to immune checkpoint blockade.

## Results

### Phosphorylation of eIF2α and cell surface expression of CRT were induced in YTN16 cells after FTD and RT treatment

**Fig. 1:**
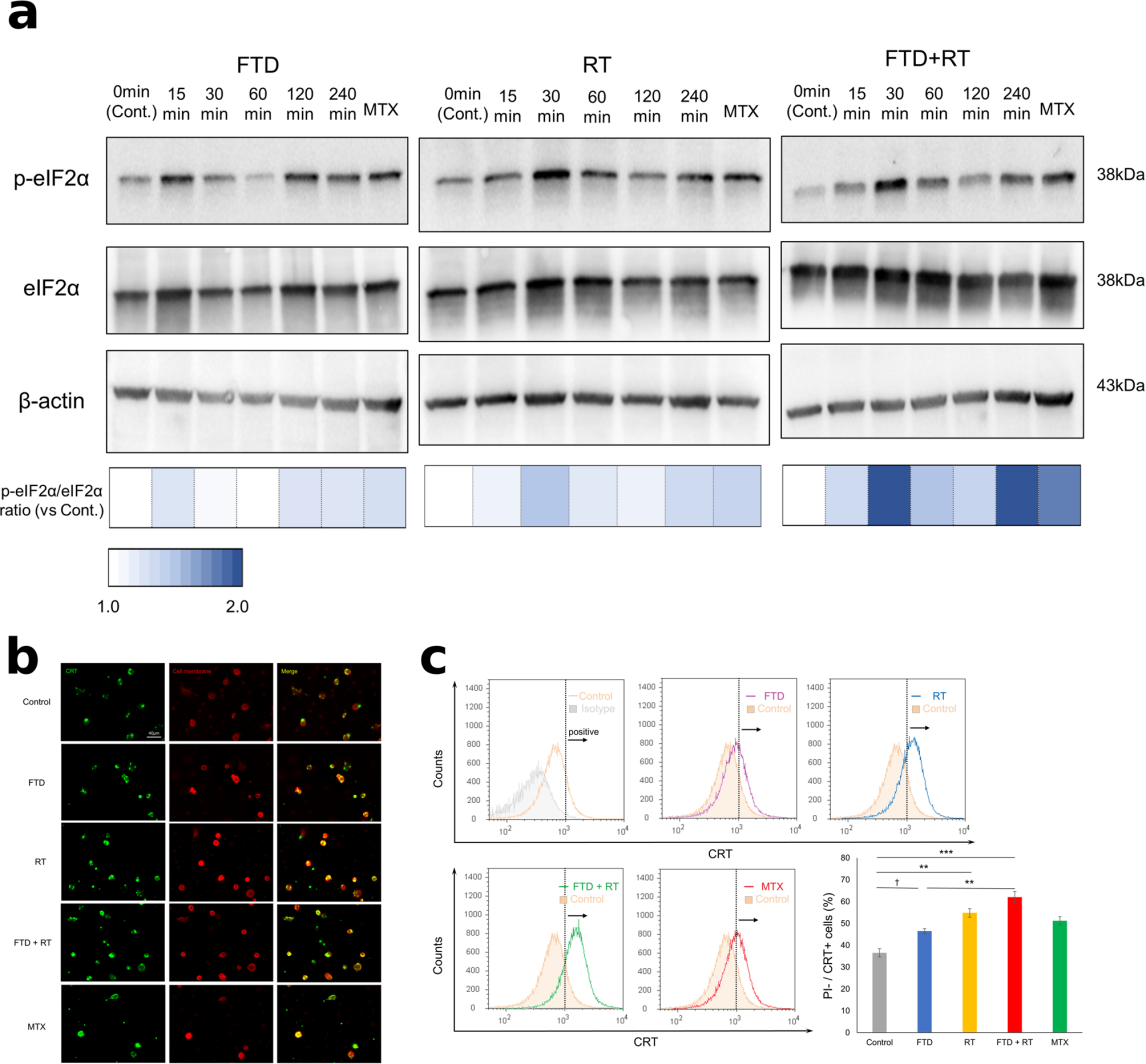
FTD and RT induce eIF2α phosphorylation and CRT exposure in YTN16 cells. YTN16 cells were treated with FTD (5 µM), RT (10 Gy), or the combination. Mitoxantrone (MTX, 1 µM) was used as a positive control. (**a**) Cells were treated as indicated for 0, 15, 30, 60, 120, and 240 min and evaluated by western blotting to assess the phosphorylation of eIF2α. (**b**) CRT expression on the cell surface was examined by immunofluorescence staining at 8 h after the indicated treatment. Green: CRT; red: cell membrane (wheat germ agglutinin); yellow: merged image. (**c**) CRT expression was quantified by flow cytometry. CRT-positive cells were defined as PI-negative events with fluorescence intensity exceeding the 99th percentile of the isotype control histogram. Data are presented as mean ± SEM from three independent experiments. Statistical analysis was performed using one-way ANOVA followed by Tukey’s HSD test (**p < 0.01, ***p < 0.001, †*p* < 0.1). Scale bars are indicated in the images. Full-length blots are provided in Supplementary Fig. S1.

We used the murine GC cell line YTN16 in this study. To evaluate the ability of FTD and RT to induce ICD in vitro, YTN16 cells were treated with FTD (5 µM) alone, RT (10 Gy) alone, or the combination. MTX (1 µM) was used as a positive control. eIF2α is a key factor in the endoplasmic reticulum (ER) stress response that promotes the translocation of CRT to the cell surface^24^. Phosphorylation of eIF2α was assessed by western blotting of YTN16 cells after treatment (Fig. 1a). In the FTD group, eIF2α phosphorylation gradually increased over time, whereas in the RT group, it peaked at 30 min and then declined. The FTD and RT combination induced stronger, more sustained eIF2α phosphorylation than either monotherapy, indicating additive effects of the combination treatment.

We next assessed ICD induction by evaluating CRT, a widely used marker of ICD, on the surface of YTN16 cells by immunofluorescence staining and flow cytometry (Fig. 1b, c). On immunofluorescence images, increased CRT signals were observed in the FTD and RT groups compared with those in the controls; higher levels were detected in the FTD + RT group compared with the single treatment groups. These results suggested enhanced CRT translocation to the cell surface after the combination treatment. The flow cytometric results were consistent with this observation. The proportion of CRT-positive cells was 36.5 ± 1.9% in the control group, 46.4 ± 1.2% in the FTD group, 54.8 ± 2.0% in the RT group, and 62.0 ± 2.6% in the FTD + RT group. The RT group showed significantly increased CRT expression compared with the control (*p* = 0.003) and the FTD group exhibited a tendency for increased CRT expression (*p* = 0.081). CRT expression was significantly higher in the FTD + RT group compared with the control (*p* < 0.001) and FTD groups (*p* = 0.009).

### FTD and RT treatment induced HMGB1 release from nuclei in YTN16 cells

**Fig. 2:**
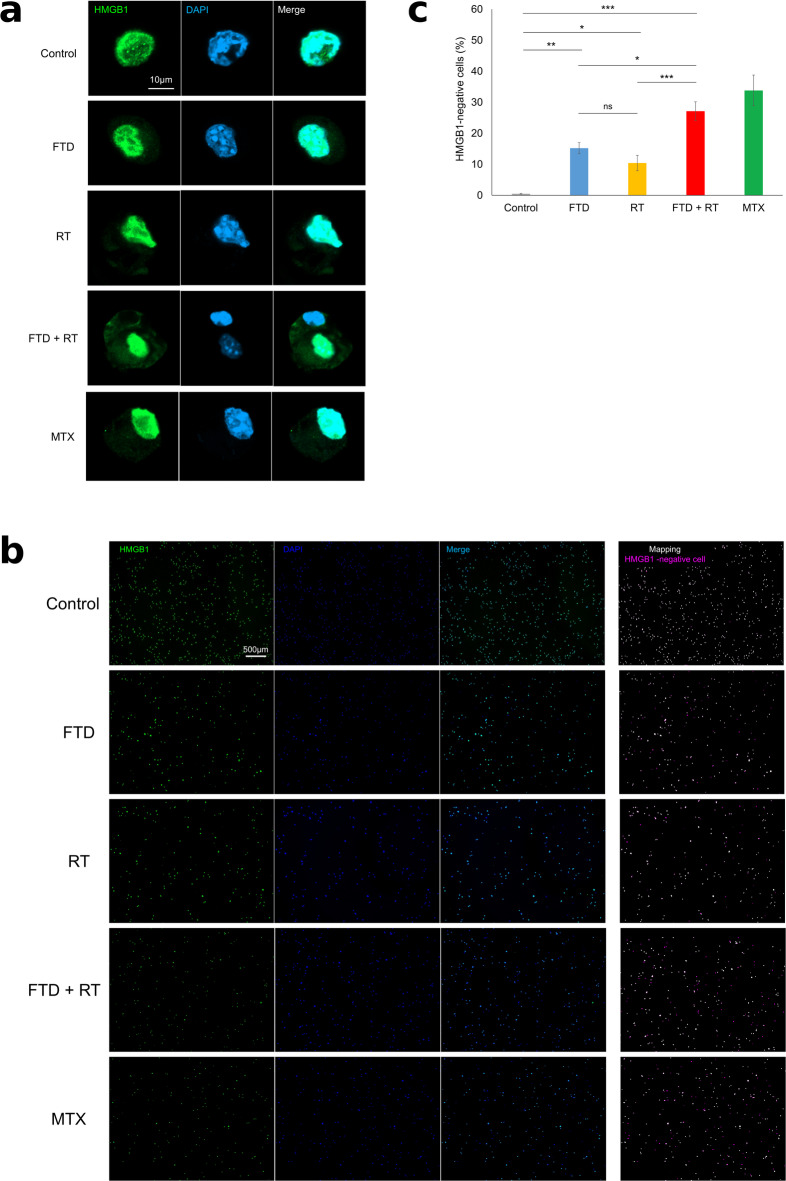
FTD and RT treatment induce HMGB1 release from nuclei of YTN16 cells. YTN16 cells were treated with FTD (5 µM), RT (10 Gy), or the combination for 24 h. MTX (1 µM) was used as a positive control. (**a**) High-magnification (×600) immunofluorescence images illustrating the localization of HMGB1 in YTN16 cells treated as indicated. Representative images are shown. (**b**) Low-magnification (×40) imaging and mapping were used to visualize HMGB1-negative cells. Cells lacking nuclear HMGB1 staining (HMGB1-negative cells) were manually identified and pseudo-colored in magenta. (**c**) The percentage of HMGB1-negative cells was calculated as the number of nuclei lacking HMGB1 staining divided by the total number of DAPI-positive nuclei per field multiplied by 100. Data are expressed as the mean ± SEM from five representative regions per experiment obtained from three independent experiments. Statistical comparisons were performed using one-way ANOVA followed by Tukey’s HSD test (*p < 0.05, **p < 0.01, ***p < 0.001, ns = not significant). Scale bars are indicated in the images.

We next assessed ICD induction by evaluating nuclear release of HMGB1, a widely used marker of ICD, by immunofluorescence staining to visualize HMGB1 localization at 24 h after treatment. A representative example illustrating HMGB1 relocalization prior to its extracellular release is shown in Fig. 2a. In untreated YTN16 cells, mostly nuclear HMGB1 staining was observed. In cells treated with FTD, RT, and the combination, HMGB1 was observed in the nucleus and cytoplasm, suggesting translocation from the nucleus after treatment.

To quantify HMGB1 loss from cells, we conducted low-magnification mapping of HMGB1-negative cells (Fig. 2b, c). HMGB1-negative cells were defined as cells with no detectable nuclear or cytoplasmic HMGB1 staining. The mean proportions of HMGB1-negative cells were 0.5 ± 0.1% in the control group, 15.2 ± 1.8% in the FTD group, 10.4 ± 2.5% in the RT group, and 27.1 ± 3.0% in the FTD + RT group. Both the FTD and RT groups showed significantly higher proportions of HMGB1-negative cells compared with the control (*p* = 0.003 and 0.045, respectively). The FTD + RT group exhibited a significantly higher proportion of HMGB1-negative cells compared with the FTD (*p* = 0.014) and RT groups (*p* < 0.001).

### FTD and RT treatment induce cytoplasmic ATP depletion in YTN16 cells

**Fig. 3:**
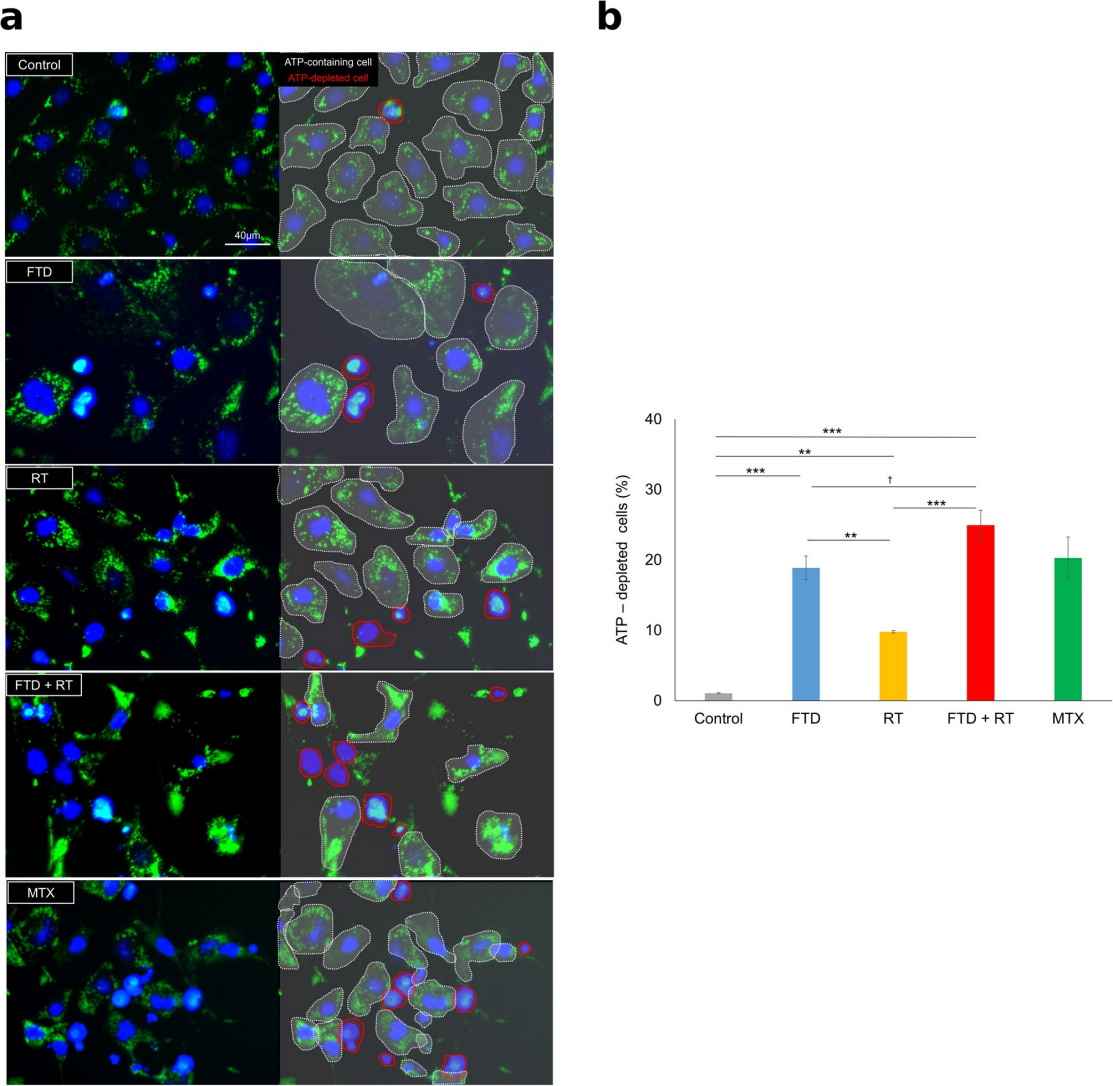
FTD and RT treatment induce cytoplasmic ATP depletion in YTN16 cells. YTN16 cells were treated with FTD (5 µM), RT (10 Gy), or the combination for 24 h. MTX (1 µM) was used as a positive control. (**a**) Representative high-magnification (×200) fluorescence microscopy images illustrating ATP levels by quinacrine staining. Quinacrine binds to cytoplasmic ATP, and ATP-depleted cells were identified by the absence of fluorescence. White dotted lines indicate ATP-positive cells, and red outlines mark ATP-depleted cells. (**b**) Quantification of ATP-depleted cells was performed by calculating the percentage of fluorescence-negative cells relative to the total number of cells per field. Data are expressed as the mean ± SEM from five representative regions per experiment, obtained from three independent experiments. Statistical comparisons were performed using one-way ANOVA followed by Tukey’s HSD test (**p < 0.01, ***p < 0.001, †*p* < 0.1). Scale bars are indicated in the images.

We further assessed ICD induction by evaluating ATP release, a representative marker of ICD. We treated cells for 24 h, stained cells with quinacrine, which binds ATP and emits fluorescence, and evaluated cells by fluorescence microscopy. An ATP-containing cell was defined as one in which fluorescence was detected in the cytoplasm surrounding the Hoechst 33342-stained nucleus; an ATP-depleted cell was defined as one in which fluorescence around the nucleus was absent (Fig. 3a). The control group included mostly ATP-containing cells, whereas the FTD and RT groups frequently showed ATP-depleted cells. The FTD + RT group exhibited qualitatively more ATP-depleted cells compared with the control, indicating enhanced cytoplasmic ATP release.

To quantify these findings, we calculated the percentage of ATP-depleted cells per field (Fig. 3b). The mean proportions of ATP-depleted cells were 1.1 ± 0.1% in the control group, 18.9 ± 1.3% in the FTD group, 9.8 ± 0.6% in the RT group, and 24.9 ± 1.1% in the FTD + RT group. All treatment groups exhibited significantly higher ATP depletion compared with the control group (FTD group, *p* < 0.001; RT group, *p* = 0.008; FTD + RT group, *p* < 0.001). The FTD + RT group showed significantly higher depletion than the RT group (*p* < 0.001) and tended to show more depletion than the FTD group (*p* = 0.052).

### Combination of FTD/TPI and RT inhibits growth of the second tumor in the mouse model

**Fig. 4:**
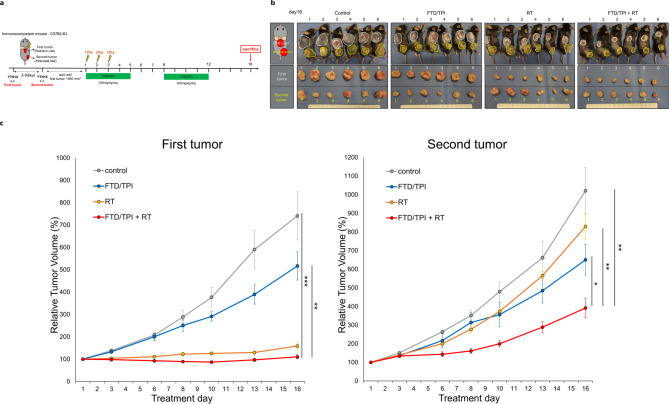
Suppression of tumor growth in a dual subcutaneous tumor mouse model by FTD/TPI and RT. (**a**) Schematic representation of the mouse model and treatment protocol. The first tumor was the irradiated site, and the second tumor served as the non-irradiated out-of-field site for evaluation of systemic antitumor effects. (**b**) Macroscopic images of the first tumors (outlined in white) and second tumors (outlined in yellow) in the indicated groups at the end of treatment (day 16) (*n* = 6 per group). (**c**) Tumor growth curves of the first and second tumors, shown as relative tumor volume (RTV). Data are presented as mean ± SEM (*n* = 6). Statistical analysis was performed using repeated-measures ANOVA, followed by Tukey’s HSD test of the RTV on day 16 (*p < 0.05, **p < 0.01, ***p < 0.001).

We next evaluated the antitumor effects of FTD/TPI, RT, and the combination in vivo using a dual subcutaneous tumor model in mice. The mouse model and treatment protocol are illustrated in Fig. 4a. At the start of treatment, tumor volumes ranged from 310.0 to 834.2 mm^3^ for the first tumor and from 47.2 to 285.8 mm^3^ for the second tumor. Baseline tumor volumes did not differ among groups (Table S1). Measurements of tumors over time in each mouse, including the shortest and longest diameters and calculated volumes, are presented in Table S2. Representative images of tumors at the end of the treatment period (day 16) are shown in Fig. 4b.

Tumor growth curves based on RTV were generated (Fig. 4c). In rmANOVA, a significant interaction between treatment group and time was observed for the first and second tumors (*p* < 0.001 for each). For the first tumor, the RTV on day 16 was 741.0 ± 107.8% in the control group, 517.1 ± 63.3% in the FTD/TPI group, 159.3 ± 17.0% in the RT group, and 110.5 ± 9.4% in the FTD/TPI + RT group. The RTV on day 16 in the FTD/TPI + RT group was significantly lower than that in the control and FTD/TPI groups (*p* < 0.001, *p* = 0.003, respectively).

For the second tumor, the RTV on day 16 was 1021.6 ± 123.6% in the control group, 650.4 ± 81.9% in the FTD/TPI group, 829.4 ± 69.3% in the RT group, and 391.8 ± 52.6% in the FTD/TPI + RT group. The FTD/TPI + RT group exhibited a significantly lower RTV of the second tumor than the other groups (control group: *p* = 0.004, FTD/TPI group: *p* = 0.039, RT group, *p* = 0.008). These results suggest that the combination of FTD/TPI and RT suppressed tumor growth in the non-irradiated second tumor more effectively than FTD/TPI or RT alone.

### FTD/TPI + RT induces ICD in the first tumor and is associated with enhanced antitumor immunity in the second tumor

**Fig. 5:**
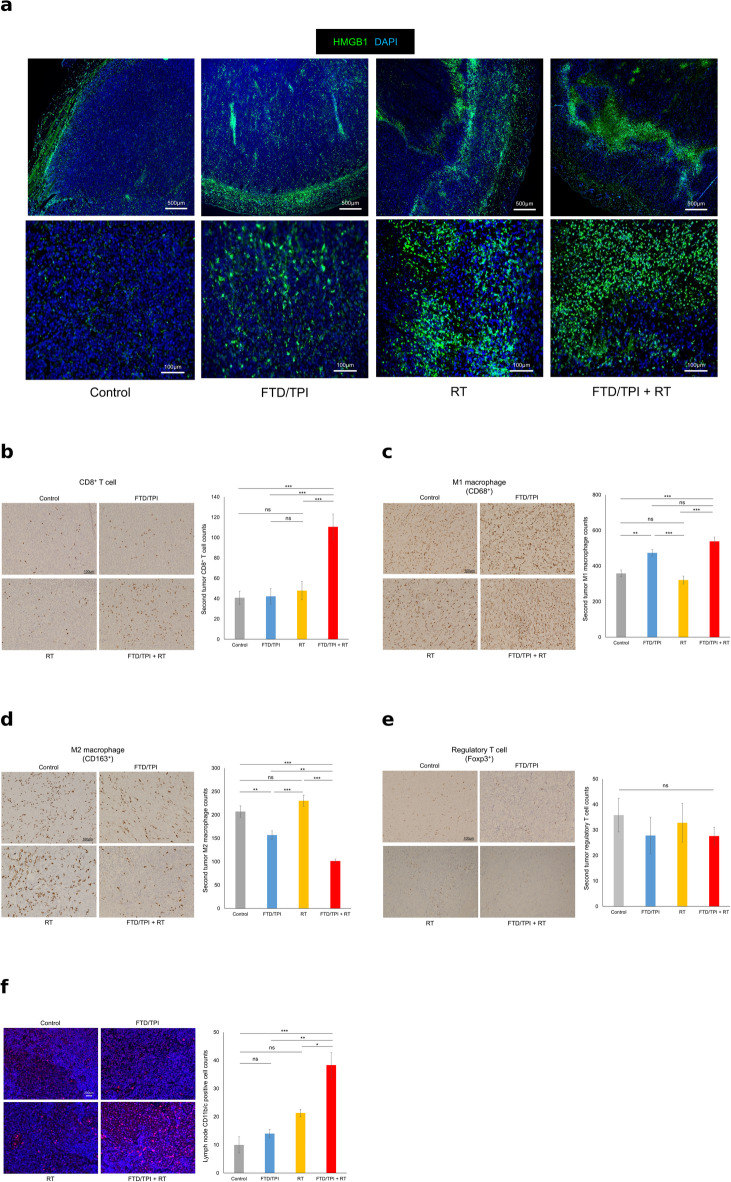
Evaluation of ICD induction in the first tumor and the immune microenvironment in the second tumor. (**a**) Immunofluorescence staining of HMGB1 in the first tumor. Green: HMGB1, blue: DAPI. Top panels show low-magnification images (×40), and bottom panels show high-magnification images (×200). (**b**–**e**) High-magnification (×100) immunohistochemical images of immune cells in the second tumor: (**b**) CD8^+^ T cells, (**c**) M1 macrophages (CD68^+^), (**d**) M2 macrophages (CD163^+^), and (**e**) regulatory T cells (Foxp3^+^). Data are expressed as the mean ± SEM from five representative regions per mouse (*n* = 6 per group). Statistical comparisons were performed using one-way ANOVA followed by Tukey’s HSD test (**p < 0.01, ***p < 0.001, ns = not significant). (**f**) High-magnification (×200) immunofluorescence images of CD11b/c⁺ dendritic cells in the subiliac lymph node draining the second tumor. Data are expressed as the mean ± SEM from five representative regions per mouse (*n* = 6 per group). Statistical comparisons were performed using one-way ANOVA followed by Tukey’s HSD test (*p < 0.05, **p < 0.01, ***p < 0.001, ns = not significant). Scale bars are indicated in the images.

To evaluate ICD induction in the first tumor, we performed immunofluorescence staining for HMGB1 in tumors at the end of treatment (day 16) (Fig. 5a). In the control group, HMGB1 was mostly confined to the nucleus with minimal extracellular staining. FTD/TPI treatment induced a broad distribution of extracellular HMGB1 signals, whereas RT treatment resulted in intense but focal release. The combination of FTD/TPI and RT produced a pattern incorporating both features, with widespread extracellular staining as well as areas of intense focal release.

To assess whether ICD in the first tumor promoted antitumor immunity, we next quantified immune cell populations in the second tumors at the end of treatment (day 16). The number of CD8⁺ T cells was significantly higher in the FTD/TPI + RT group than in other groups (all *p* < 0.001) (Fig. 5b). The number of M1 macrophages (CD68^+^) was significantly higher in the FTD/TPI (*p* = 0.005) and FTD/TPI + RT groups (*p* < 0.001) compared with the control group; the FTD/TPI and FTD/TPI + RT groups also showed significantly higher numbers of M1 macrophages compared with the RT group (both *p* < 0.001) (Fig. 5c).

The number of M2 macrophages (CD163^+^) was significantly reduced in both the FTD/TPI (*p* = 0.009) and FTD/TPI + RT groups (*p* < 0.001) compared with the control group (Fig. 5d). The FTD/TPI + RT group showed significantly fewer M2 macrophages than the FTD/TPI group (*p* = 0.004). The FTD/TPI and FTD/TPI + RT groups showed significantly fewer M2 macrophages compared with the RT group (both *p* < 0.001); no significant difference was observed between the RT and control groups (*p* > 0.05).

No significant differences were observed in the number of regulatory T cells (Foxp3^+^) among the groups (all pairwise comparisons, *p* > 0.05) (Fig. 5e).

To investigate whether immune activation extended beyond the tumor microenvironment, we analyzed the number of CD11b/c⁺ dendritic cells infiltrating the subiliac lymph nodes draining the second tumor. The FTD/TPI + RT group exhibited significantly higher CD11b/c⁺ cell counts compared with the control (*p* < 0.001), FTD/TPI (*p* = 0.0013), and RT (*p* = 0.012) groups (Fig. 5f). No significant differences were observed in the other pairwise comparisons.

### PD-1 blockade further enhances the antitumor effect of FTD/TPI + RT in mice

**Fig. 6:**
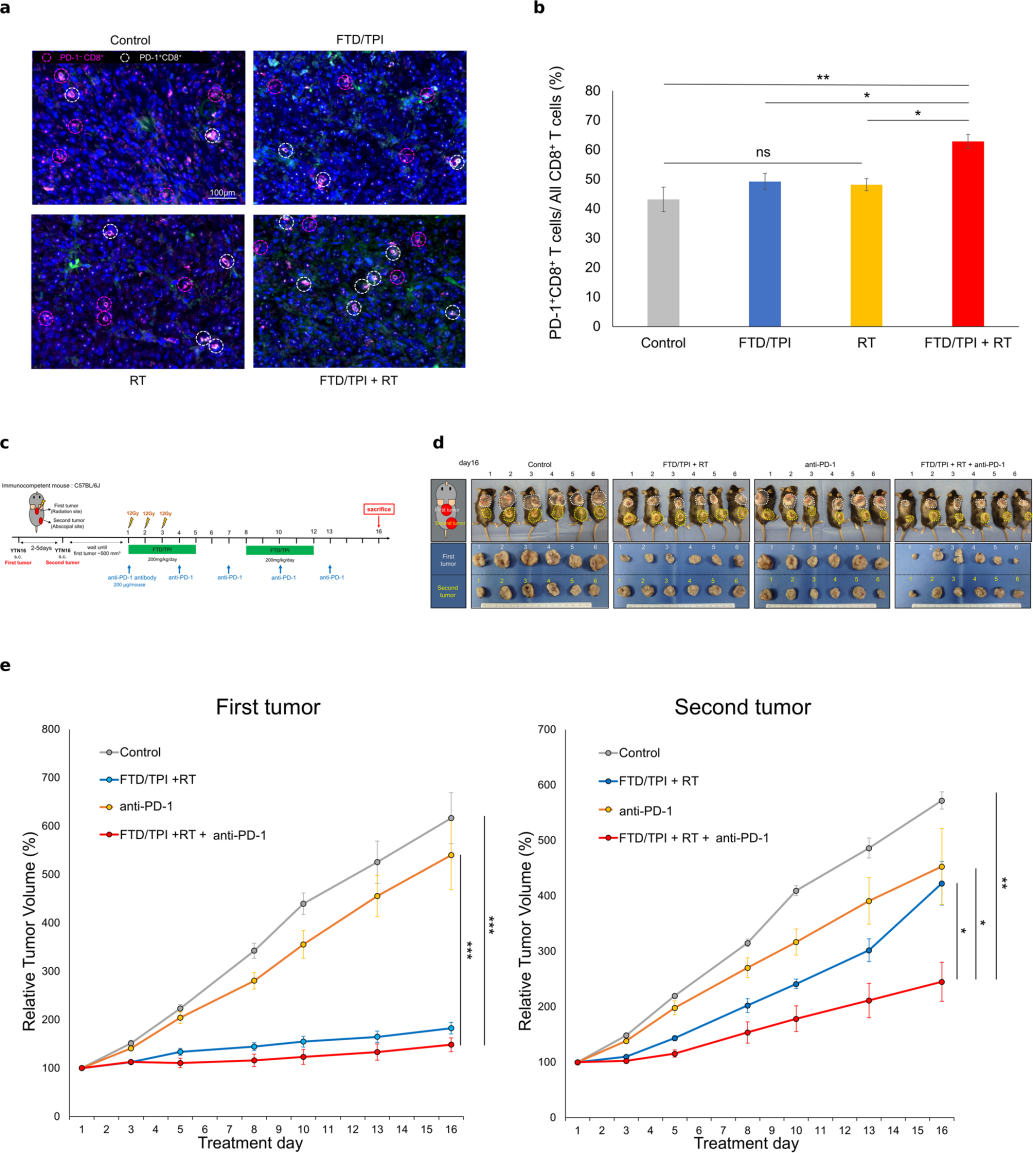
Enhancement of tumor growth inhibition by anti-PD-1 antibody with FTD/TPI and RT. (**a**) High-magnification (×200) dual-immunofluorescence images showing PD-1 expression in CD8⁺ T cells infiltrating the second tumor. Pink circles indicate PD-1⁻CD8⁺ T cells, and white circles indicate PD-1⁺CD8⁺ T cells. (**b**) Percentage of PD-1⁺CD8⁺ T cells calculated relative to the total CD8⁺ T cells per field. (**c**) Schematic overview of the dual tumor mouse model treated with anti-PD-1 antibody in addition to FTD/TPI and RT. (**d**) Macroscopic images of the first tumor (outlined in white) and second tumor (outlined in yellow) in the indicated groups at the end of treatment (day 16) (*n* = 6 per group). (**e**) Tumor growth curves of the first and second tumors, shown as RTV. Data are presented as mean ± SEM (*n* = 6). Statistical comparisons: (**b**) one-way ANOVA with Tukey’s HSD; (**e**) repeated-measures ANOVA with Tukey’s HSD on day 16 (*p < 0.05, **p < 0.01, ***p < 0.001, ns = not significant). Scale bars are indicated in the images.

We evaluated PD-1 expression on CD8⁺ T cells in the second tumor using dual-fluorescent immunostaining (Fig. 6a). The proportion of PD-1⁺CD8⁺ cells among total CD8⁺ T cells was 43.2 ± 3.4% in the control group, 49.2 ± 2.3% in the FTD/TPI group, 48.2 ± 1.7% in the RT group, and 62.9 ± 1.9% in the FTD/TPI + RT group. The FTD/TPI + RT group showed a significantly higher proportion of PD-1⁺CD8⁺ T cells compared with the control (*p* = 0.007), FTD/TPI (*p* = 0.046), and RT (*p* = 0.032) groups; no significant differences were observed among the other groups (Fig. 6b). These data indicate increased PD-1 expression on CD8⁺ T cells after FTD/TPI + RT. While PD-1 expression alone does not distinguish activation from functional exhaustion, this result supports further evaluation of anti-PD-1 therapy in this setting.

We next evaluated the effect of PD-1 blockade combined with the FTD/TPI + RT combination treatment on tumor progression in the dual subcutaneous tumor mouse model (Fig. 6c). At treatment initiation, the volume of the first tumor ranged from 250.3 to 752.6 mm^3^, while the volume of the second tumor ranged from 227.9 to 463.9 mm^3^. Baseline tumor volumes did not differ among groups (Table S3). Measurements of tumors over time in each mouse, including the shortest and longest diameters and calculated volumes, are presented in Table S4. Images of tumors at the end of the treatment (day 16) are presented in Fig. 6d.

RTV-based tumor growth curves are shown in Fig. 6e. Based on the rmANOVA, a significant interaction between treatment group and time was observed for the first and second tumors (*p* < 0.001, *p* = 0.002, respectively). In the first tumor, the RTV on day 16 was 616.5 ± 52.7% in the control group, 540.2 ± 71.7% in the anti-PD-1 group, 182.6 ± 12.0% in the FTD/TPI + RT group, and 148.4 ± 14.2% in the FTD/TPI + RT + anti-PD-1 group. The RTV in the FTD/TPI + RT and FTD/TPI + RT + anti-PD-1 groups was significantly smaller than that in the control and anti-PD-1 groups (*p* < 0.001 for each comparison).

In the second tumor, the RTV values on day 16 were 572.2 ± 44.5% in the control group, 453.1 ± 68.9% in the anti-PD-1 group, 422.6 ± 39.3% in the FTD/TPI + RT group, and 245.1 ± 35.1% in the FTD/TPI + RT + anti-PD-1 group. The RTV in the FTD/TPI + RT + anti-PD-1 group was significantly lower than in the control (*p* = 0.001), anti-PD-1 (*p* = 0.02), and FTD/TPI + RT (*p* = 0.04) groups. This result suggests that PD-1 blockade further enhanced the tumor-suppressive effect of FTD/TPI + RT at the non-irradiated second tumor site.

Notably, across both in vivo experiments, all animals completed the protocol and no expected or unexpected adverse events were observed.

## Discussion

In the present study, we showed that FTD/TPI markedly enhanced RT-induced ICD and systemic antitumor immune responses in GC in a dual subcutaneous tumor mouse model. In addition to direct tumor growth inhibition, the FTD/TPI and RT combination promoted dendritic cell activation, increased CD8⁺ T-cell responses, and improved sensitivity to PD-1 blockade in the non-irradiated second tumor, highlighting its potential as a novel radio-chemoimmunotherapy strategy. Notably, this immunogenic effect contrasts with that of conventional pyrimidine antimetabolites, such as 5-fluorouracil, one of the key agents in GC treatment, which are not among the well-established inducers of ICD^7–10^. This highlights the unique immunomodulatory properties of FTD/TPI.

Mechanistically, we demonstrated that FTD/TPI induces ICD in murine YTN16 GC cells, as evidenced by eIF2α phosphorylation, increased CRT surface exposure, and enhanced release of HMGB1 and ATP. As a marker of ER stress responses, eIF2α phosphorylation has emerged as a pathognomonic feature of ICD, showing a strong correlation with CRT exposure and downstream antitumor immune activation^25^. Systematic analyses have shown that other canonical ER stress markers, such as activating transcription factor 4 (ATF4) activation and X-box binding protein 1 (XBP1) gene splicing, are not consistently associated with ICD, underscoring the unique relevance of eIF2α phosphorylation in this context. Notably, combined treatment with FTD/TPI and RT further amplified eIF2α phosphorylation compared with single treatments, reinforcing its role as a key mediator linking DNA damage to immunogenic signaling. FTD/TPI has also been reported to enhance radiosensitivity through sustained DNA damage and S-phase arrest, suggesting it may amplify RT-induced ICD through complementary mechanisms^26^. Additionally, prior studies in other tumor types support the biological plausibility of our findings. In a murine colorectal cancer model, Limagne et al. showed that FTD/TPI-based treatment induced hallmarks of immunogenic cell death, depleted immunosuppressive macrophages, and enhanced the efficacy of PD-1 blockade^23^. Although evidence in GC remains limited, these findings collectively support the immunogenic and therapeutic relevance of FTD/TPI-based combinations beyond a single model. To our knowledge, our findings represent the first preclinical demonstration that FTD/TPI and RT exert additive effects on ICD induction, highlighting the mechanistic novelty of this combination.

Notably, robust ICD induction by combined FTD/TPI and RT in the mouse model was accompanied by increased accumulation of dendritic cells in tumor-draining lymph nodes, indicating enhanced antigen presentation and effective priming of tumor-specific T cells. This finding positions dendritic cell activation as an immediate downstream consequence of ICD, linking tumor cell stress to adaptive immune engagement.

We also observed that ICD induction was associated with marked remodeling of the tumor immune microenvironment in the second, non-irradiated tumor. Notably, FTD/TPI, particularly in combination with RT, significantly suppressed infiltration of CD163⁺ M2 macrophages, which promote immune evasion and tumor progression. Previous studies reported that FTD treatment increased the secretion of inflammatory cytokines such as IL-2, GM-CSF, IFN-γ, and TNF-α in a co-culture model of cancer cells and peripheral blood mononuclear cells^27^. This cytokine profile is consistent with an M1-promoting immune environment. Additionally, DAMPs released during ICD may activate innate immune pathways, potentially including cGAS–STING signaling^28^, thereby contributing to macrophage reprogramming. While the precise regulatory mechanisms remain to be elucidated, these findings suggest that ICD induction by FTD/TPI reshapes the tumor immune milieu in a manner favorable to antitumor immunity.

As a downstream consequence of enhanced antigen presentation and remodeling of the immunosuppressive tumor microenvironment, FTD/TPI + RT increased PD-1 expression on tumor-infiltrating CD8⁺ T cells, likely reflecting heightened antigen exposure and T-cell activation. Moreover, the addition of anti-PD-1 therapy to FTD/TPI + RT further suppressed second tumor growth. Together, these findings provide proof-of-concept evidence that FTD/TPI + RT may create an immune context in which PD-1 blockade becomes more effective, providing a biological rationale for further evaluation of this combination.

In the present model, only the first tumor was directly irradiated, while the second tumor, located on the contralateral flank, was positioned outside the radiation field to evaluate systemic antitumor effects. Although complete exclusion of low-dose scatter radiation or short-range bystander effects cannot be guaranteed, several observations in this study support a predominantly immune-mediated mechanism for the observed out-of-field tumor suppression. Specifically, increased dendritic cell accumulation in the draining lymph nodes of the second tumor, enhanced infiltration of CD8⁺ T cells, and responsiveness to anti-PD-1 therapy are features not readily accounted for by local bystander effects, which typically act over shorter distances through non-immune paracrine signaling. These findings are therefore consistent with an abscopal-like systemic antitumor response, although strict distinction from local bystander effects warrants further investigation.

ICIs combined with chemotherapy are the current first-line treatment for unresectable, HER2-negative GC^29,30^. However, the benefit of these regimens is limited in patients with low PD-L1 expression. Our findings suggest that the combination of FTD/TPI and RT may enhance tumor immunogenicity and reshape the tumor immune microenvironment, thereby sensitizing tumors to PD-1 blockade even in immunotherapy-refractory settings. This strategy may expand the population of patients who may benefit from immunotherapy-based approaches.

This study has several limitations. First, experiments were performed using a single syngeneic murine GC cell line, YTN16, reflecting the limited availability of immunocompetent GC models. Second, the hypofractionated RT regimen used in this study, while supported by preclinical evidence of immunogenicity, does not reflect standard clinical practice. Third, the methodological characterization of ICD was limited: HMGB1 and ATP release were assessed by fluorescence-based approaches rather than direct extracellular quantification (e.g., ELISA or luciferin/luciferase assay), and quinacrine staining, in particular, may be affected by lysosomal pH or drug efflux. These findings should therefore be interpreted together with the other ICD markers evaluated in this study. Immune profiling also used a limited set of markers, precluding definitive conclusions regarding the functional status of tumor-infiltrating immune cells. Fourth, the PD-1 blockade experiment has interpretive limitations: PD-1 expression alone cannot distinguish T-cell activation from functional exhaustion^31^, and additional exhaustion markers (e.g., TIM-3, LAG-3, TOX) and functional assays were not evaluated. Moreover, the triple-combination experiment did not include an RT plus anti-PD-1 comparator, precluding determination of the incremental contribution of FTD/TPI beyond RT + anti-PD-1. Fifth, survival, body weight, and serum biochemistry were not prospectively collected, as the in vivo experiments were designed with a predefined day 16 endpoint; although all animals completed the protocol without observable adverse events, systematic safety and survival evaluation was not performed. Future studies should address these limitations through several directions. Validation in additional GC models and clinically applicable RT schedules, including both conventional fractionation and stereotactic-type hypofractionated approaches with appropriate comparator groups such as RT plus anti-PD-1, will be essential to establish generalizability and translational relevance. Such studies should also incorporate comprehensive functional immune analyses, including exhaustion markers, as well as systematic safety and survival evaluation. Collectively, these investigations will help define the mechanisms, optimal conditions, and clinical applicability of FTD/TPI-based radio-chemoimmunotherapy for promoting systemic antitumor immunity, particularly in patients with immunotherapy-refractory disease.

In conclusion, this study demonstrates that FTD/TPI enhances RT-induced ICD, actively remodels the immunosuppressive tumor microenvironment, and augments the efficacy of PD-1 blockade, resulting in a potent abscopal effect in GC. These findings provide a strong mechanistic and translational rationale for clinical trials evaluating FTD/TPI-based radio-chemoimmunotherapy, particularly in patients with limited responsiveness to ICIs.

## Methods

### Cell lines and cell culture

The murine YTN16 cell line, derived from C57BL/6 mice, was used as a syngeneic model for GC and kindly provided by a coauthor (Dr. Sachiyo Nomura, Hoshi University, Tokyo, Japan). The YTN16 cell line was originally established and characterized by Yamamoto et al.^32^ as part of the YTN series of murine GC cell lines and has since been maintained in our laboratory. Cells were cultured in high-glucose Dulbecco’s modified Eagle medium (DMEM, Sigma-Aldrich, Saint Louis, MO, USA) containing 0.1% MITO (Corning Japan, Tokyo, Japan), 1% L-glutamine, 1% penicillin/streptomycin, and 10% FBS in plastic dishes coated with Type I collagen solution (0.5% Atelocollagen Acidic Solution, IPC-50; Koken Co., Ltd., Tokyo, Japan) at 37 °C in a 5% CO_2_ atmosphere. The YTN16 line is one of the few murine GC cell lines that reproducibly forms tumors in immunocompetent C57BL/6J mice^32^. This syngeneic combination allows for the evaluation of tumor–immune interactions in a physiologically relevant and immunologically intact setting^33–35^.

### Chemicals and cell treatments

FTD and TPI were obtained from FUJIFILM Wako Chemical Corporation (Osaka, Japan) and Fluorochem Ltd. (Hadfield, UK), respectively. Mitoxantrone (MTX) was obtained from LKT Labs, Inc. (St Paul, MN, USA) and diluted in DMEM for the in vitro experiments.

Cells were irradiated with 10 Gy using an X-ray irradiator (MBR-1520R; Hitachi, Ibaraki, Japan). The irradiated cells were then refreshed with medium containing 5 µM FTD and 1 µM MTX.

### Western blotting analysis

Cells were harvested at 0 (control), 15, 30, 60, 120, and 240 min after treatment. Whole cell lysates were prepared using RIPA buffer (FUJIFILM Wako Chemical Corporation, Cat# 188–02453) supplemented with Protease Inhibitor Cocktail (Sigma-Aldrich, Cat# P1860-1ML) and Phosphatase Inhibitor Cocktail 3 (Sigma-Aldrich, Cat# P0044-1ML). Protein concentrations were determined using the BCA Protein Assay Kit (Thermo Fisher Scientific, Waltham, MA, USA, Cat# 23227). Equal amounts of protein were separated by SDS-PAGE on 10% Mini-PROTEAN^®^ TGX™ precast gels (Bio-Rad, Hercules, CA, USA, Cat# 4561035) and transferred to PVDF membranes using the Trans-Blot^®^ Turbo Transfer System with Mini PVDF Transfer Packs (Bio-Rad, Cat# 1704156). Membranes were blocked with blocking solution (0.1% Tween-20; EZ Block, ATTO Corporation, Tokyo, Japan). The following primary antibodies were used for immunoblotting: eIF2α (D7D3, rabbit monoclonal IgG; Cell Signaling Technology, Danvers, MA, USA, Cat# 5324; dilution 1:1000) and Phospho-eIF2α (D9G8, rabbit monoclonal IgG; Cell Signaling Technology, Cat# 3398; dilution 1:1000). The β-actin monoclonal antibody (AC-15, mouse IgG; Sigma-Aldrich, Cat# A5441; dilution 1:5000) was used as a loading control. The membranes were subsequently incubated with appropriate horseradish peroxidase (HRP)-conjugated secondary antibodies for 1 h, and bands were visualized using Amersham™ ECL™ Prime Western Blotting Detection Reagent (GE Healthcare/Cytiva, Tokyo, Japan, Cat# RPN2232) with the Light-Capture system (ATTO Corporation, Tokyo, Japan). Protein expression levels were quantified using the CS Analyzer system (ATTO Corporation) and normalized to the 0-min control.

### Flow cytometric analysis

Cells were harvested at 8 h after treatment. After washing with phosphate buffered saline (PBS), the cells were fixed with 0.25% paraformaldehyde for 5 min. The cells were then incubated with a rabbit anti-CRT antibody (ab2907; Abcam, Cambridge, UK; dilution 1:200) diluted in staining buffer (2% fetal bovine serum in PBS) for 30 min. An isotype control antibody (Rabbit mAb IgG XP^®^ Isotype Control; Cell Signaling Technology, Cat# 3900; dilution 1:1000) was used in parallel. After washing, the cells were incubated with Alexa Fluor^®^ 488-conjugated goat anti-rabbit IgG antibody (Invitrogen, Carlsbad, CA, USA, Cat# A-11008; dilution 1:500) diluted in staining buffer for 30 min. Propidium iodide (final concentration 1 µg/ml) was used to stain dead cells. Flow cytometric analysis was carried out using the first‑generation Attune™ Acoustic Focusing Cytometer (Thermo Fisher Scientific). The positivity rate of CRT was quantified by counting viable cells (propidium iodide–negative cells) with fluorescence intensity exceeding the 99th percentile of the isotype control histogram.

### Immunofluorescence staining of cells

Immunofluorescence staining was performed using the antibodies used for flow cytometry. After washing with PBS, the cells were fixed in 4% paraformaldehyde for 15 min. The cells were then incubated overnight with rabbit anti-CRT primary antibody (dilution 1:200) and subsequently incubated with Alexa Fluor^®^ 488-conjugated goat anti-rabbit IgG secondary antibody (dilution 1:500) at room temperature for 1 h. The cell membrane was stained with Wheat Germ Agglutinin CF^®^ 633 Conjugates (Biotium, Fremont, CA, USA, Cat# 29024; final concentration 5 µg/mL) for 20 min, and the cells were then mounted. Fluorescence images were acquired using an all-in-one fluorescence microscope (BZ-X800; KEYENCE, Osaka, Japan).

### HMGB1 release

We evaluated the extracellular release of HMGB1 at 24 h after treatment using immunofluorescence staining. The cells were washed with PBS, fixed with 4% paraformaldehyde for 5 min, and permeabilized with 0.01% Triton X-100 for 15 min. After washing, cells were incubated overnight with rabbit anti-HMGB1 antibody (Abcam, Cat# ab227168; dilution 1:1000) and then incubated for 1 h with Alexa Fluor^®^ 488-conjugated goat anti-rabbit IgG antibody (dilution 1:500). After nuclear staining with 2 µM 4′,6-diamidino-2-phenylindole for 20 min, the cells were mounted. Fluorescence images were acquired using the all-in-one fluorescence microscope for low-magnification imaging (×40) and mapping, and a confocal laser microscope (LSM980; ZEISS, Oberkochen, Germany) was used for high-magnification imaging (×600). The percentage of HMGB1-negative cells was calculated as the number of nuclei lacking HMGB1 staining divided by the total number of DAPI-positive nuclei per field multiplied by 100.

### ATP release

The release of ATP into the extracellular space was assessed at 24 h after treatment. Quinacrine was used to label intracellular ATP; it binds to ATP in cells and emits fluorescence, which confirms the presence of intracellular ATP. The cells were incubated with 5 µM quinacrine (Abcam, Cat# ab120749) and 1 µg/mL Hoechst 33342 in Krebs-Ringer solution (125 mM NaCl, 5 mM KCl, 1 mM MgSO_4_, 0.7 mM KH_2_PO_4_, 2 mM CaCl_2_, 6 mM glucose, and 25 mM HEPES, pH 7.4) at 37 °C for 30 min. The cells were observed under an all-in-one fluorescence microscope at ×200 magnification.

An ATP-containing cell was defined as one in which fluorescence was detected in the cytoplasm surrounding the Hoechst 33342-stained nucleus; an ATP-depleted cell was defined as one in which fluorescence around the stained nucleus was absent. The percentage of ATP-depleted cells was calculated as the number of cells lacking cytoplasmic quinacrine fluorescence divided by the total number of Hoechst 33342-positive nuclei per field multiplied by 100.

### Mouse model

The animal use proposal and experimental protocol (AP-204184) were reviewed and approved by the Animal Care and Use Committee of the Kanazawa University. All animal experiments were performed following the standard guidelines of Kanazawa University. This study was reported in accordance with the ARRIVE guidelines. C57BL/6J mice (wild-type) were purchased from Charles River Laboratories, Inc. (Wilmington, MA, USA) and acclimatized for 7 days after arrival. The mice were housed under standard conditions at 22–24 °C and 40%–60% relative humidity on a 12:12 h light–dark cycle, with food and water provided ad libitum; no special environmental enrichment was used.

We generated the model bearing two subcutaneous tumors using female mice at 6–7 weeks of age; animals were immunocompetent, and no procedures were performed prior to tumor cell implantation. YTN16 cells (2.0 × 10^6^ cells suspended in 200 µL of high-glucose DMEM) were injected subcutaneously into the midline of the dorsal back under anesthesia induced by intraperitoneal injection of a mixed anesthetic (medetomidine 0.3 mg/kg, midazolam 4.0 mg/kg, and butorphanol 5.0 mg/kg; hereafter referred to as MMB anesthesia) to establish the first tumor. This combined MMB anesthesia regimen has been widely used in mice, and its efficacy and safety have been documented^36^. After 2–5 days, the same number of cells was injected into the left flank to generate the second tumor. The first tumor was the target of local irradiation, whereas the second tumor was positioned outside the radiation field and used to evaluate systemic antitumor effects at a non-irradiated site. Tumor growth was monitored every 2–3 days. Following our institutional animal care guidelines, mice were euthanized if tumors exceeded a maximum diameter of 20 mm or if they exhibited signs of distress. Predefined exclusion criteria were ulceration or necrosis precluding reliable caliper measurement, non-protocol-related death, or protocol deviations (e.g., mistimed dosing/irradiation). No outlier removal was planned. All animals and data points met the prespecified criteria, and no animals or measurements were excluded.

### Animal grouping and treatment

Mice were randomly allocated to treatment groups using a simple randomization method (drawing lots); the randomization sequence was generated by a computer random-number generator. Potential confounders (cage location, handling/measurement order, time of day) were not systematically controlled; measurements were performed at consistent times of day by the same operator. A total of 24 mice were allocated into the Control, FTD/TPI, RT, and FTD/TPI + RT groups (*n* = 6/group). The group size was prespecified on the basis of feasibility considerations and to minimize animal use; no formal a priori power calculation was performed. Treatment was initiated when the volume of the first tumor reached approximately 500 mm^3^. The two tumors were not intended to be size-matched at treatment initiation, because they served different experimental purposes: the first tumor was used as the local irradiation target, whereas the second tumor was used to evaluate systemic antitumor effects at a non-irradiated site. Accordingly, the second tumor was smaller at baseline. Similar asymmetric dual-tumor designs have been used in previous preclinical studies of radiation-induced out-of-field or abscopal responses^37^. Tumor volume was calculated using the formula:

$$\text{Tumor volume}=(L\times W^{2})/2,$$

where L and W are the longest and shortest tumor diameters, respectively.

Radiation at a dose of 12 Gy was administered using the X-ray irradiator on days 1, 2, and 3. To ensure localized irradiation of only the first tumor, mice were anesthetized and most of the body was covered with 3-mm lead shielding, leaving only the first tumor exposed to the radiation field. FTD/TPI was administered orally on days 1–5 and 8–12. The drugs were dissolved in carboxymethyl cellulose at a molar ratio of 1:0.5 and administered at a dose of 200 mg/kg/day, calculated based on the amount of FTD. This dosing regimen was adopted from a previous preclinical study demonstrating the antitumor efficacy of FTD/TPI in murine models^38^. Using standard body surface area conversion, this dose corresponds to approximately 16 mg/m^2^/day in humans, which is lower than the clinical dose of 70 mg/m^2^/day used in FTD/TPI therapy for GC^15^. Thus, the dosage used in this study falls within a translationally relevant and pharmacologically feasible range. The mice were sacrificed on day 16. Euthanasia was performed by cervical dislocation under MMB anesthesia; death was confirmed by the absence of respiration, heartbeat, and reflex responses, in accordance with the AVMA Guidelines for the Euthanasia of Animals (2020)^39^. Tumors were excised and examined using immunohistochemistry and immunofluorescence. Subiliac lymph nodes were also excised and evaluated.

In other experiments, 24 mice were allocated to the four groups: control, anti-PD-1, FTD/TPI + RT, and FTD/TPI + RT + anti-PD-1 (*n* = 6/group). RT and FTD/TPI were delivered as described above. Anti-PD-1 antibody (Ono Pharmaceutical Co., Ltd., Osaka, Japan) was administered intraperitoneally at a dose of 200 µg/mouse on days 1, 4, 7, 10, and 13. Tumor volume was calculated using the formula described above.

The total number of animals used was 48. All mice completed the protocol; no animals were lost or excluded.

### Immunohistochemistry

Tumor specimens were fixed in 10% neutral-buffered formalin and embedded in paraffin. Deparaffinized sections were pretreated by autoclaving in 10% citric acid buffer at 120 °C for 15 min. After treatment with protein block serum (Dako Co., Kyoto, Japan) for 10 min and incubation with 2% skim milk for 30 min to block nonspecific reactions, sections were incubated with primary antibody at 4 °C overnight. The following primary antibodies were used: CD8 (Abcam, Cat# ab209775; dilution 1:2000) for CD8⁺ T cells, CD68 (Abcam, Cat# ab125212; dilution 1:1000) for M1 macrophages, CD163 (Abcam, Cat# ab182422; dilution 1:500) for M2 macrophages, and Foxp3 (Novus Biologicals, Centennial, CO, USA, Cat# NB600-245; dilution 1:200) for regulatory T cells. Sections were examined using an Olympus light microscope (Olympus Corporation, Tokyo, Japan) equipped with a UPLFLN 100× objective lens, DP70-SET microscope digital camera, and cellSens v1.16 as the acquisition software; images were obtained at ×100 magnification.

### Immunofluorescence of tissue sections

Immunofluorescence staining was performed using paraffin-embedded sections processed as described in the immunohistochemistry section. The first tumors were stained with rabbit anti-HMGB1 antibody (dilution 1:1000) and Alexa Fluor^®^ 488-conjugated goat anti-rabbit IgG antibody (dilution 1:500). CD11b/c^+^ dendritic cells in subiliac lymph nodes were detected using an anti-CD11b/c antibody (Novus Biologicals, Cat# NB110-40766; dilution 1:200) and Alexa Fluor^®^ 647-conjugated goat anti-rabbit IgG (Abcam, Cat# ab150079; dilution 1:500). For dual-fluorescence immunostaining, PD-1 expression on CD8⁺ T cells was detected using rabbit anti-PD-1 antibody (Abcam, Cat# ab214421; dilution 1:100) and Alexa Fluor^®^ 488-conjugated goat anti-rabbit IgG (dilution 1:500). After washing and blocking, sections were incubated with an Alexa Fluor^®^ 647-conjugated anti-CD8 antibody (Abcam, Cat# ab225491; dilution 1:1000). Nuclei were counterstained with 2 µM DAPI, and fluorescence images were captured using the all-in-one fluorescence microscope (magnifications are described in the figure legends).

### Statistical analysis

Statistical analyses were performed using SPSS software version 23 (IBM Corp., Armonk, NY, USA). Group comparisons were performed using one-way analysis of variance (ANOVA) after confirming normality (Shapiro–Wilk) and homogeneity of variances (Levene); post hoc pairwise comparisons used Tukey’s honestly significant difference test. In the in vivo experiments, tumor response of the second tumor was evaluated using the relative tumor volume. Baseline tumor volumes (first day of treatment) were compared across groups. Subsequently, tumor growth was expressed as relative tumor volume (normalized to baseline, set to 100%) and analyzed using repeated-measures ANOVA to assess the interaction between treatment group and time. When a significant interaction was detected, Tukey’s honestly significant difference test was applied for pairwise comparisons on the final day of treatment. *p* < 0.05 indicated statistical significance. Results are presented as mean ± SEM. All in vitro experiments were repeated independently three times. Each data point in the microscopy-based cell counting analyses was obtained by averaging values from five fields per sample. The experimental unit was the mouse for in vivo analyses (microscopy counts were averaged to one value per mouse) and the culture dish for in vitro assays. Group allocation was known during allocation and treatment. Outcome assessment could not be blinded because the treatment procedures (e.g., unilateral irradiation with shielding and dosing schedules) made group identity evident at the time of measurement. Data analysis was performed using coded IDs and predefined quantitative criteria to minimize bias.

## Supplementary Information

Below is the link to the electronic supplementary material.

Supplementary Material 1

## Data Availability

The datasets used and/or analyzed during the current study are available from the corresponding author on reasonable request.
