# Supplementary material for: Trifluridine/tipiracil enhances radiation-induced abscopal effects and augments PD-1 blockade in gastric cancer
Source: Sci Rep. 2026 May 21;16:23124. doi: 10.1038/s41598-026-53689-9 (PMC13396637; doi:10.1038/s41598-026-53689-9)
Supplement: Supplementary file 1 — Supplementary Material 1 [file 41598_2026_53689_MOESM1_ESM.pdf]

Supplementary Figure S1

Related to Fig. 1a.

Full-length (uncropped) membranes corresponding to the immunoblots shown in Fig. 1a (eIF2α, phospho-eIF2α, and β-actin) under the indicated conditions (FTD, RT, and FTD+RT). Blots were cropped for presentation in the main figure.

FTD

eIF2α

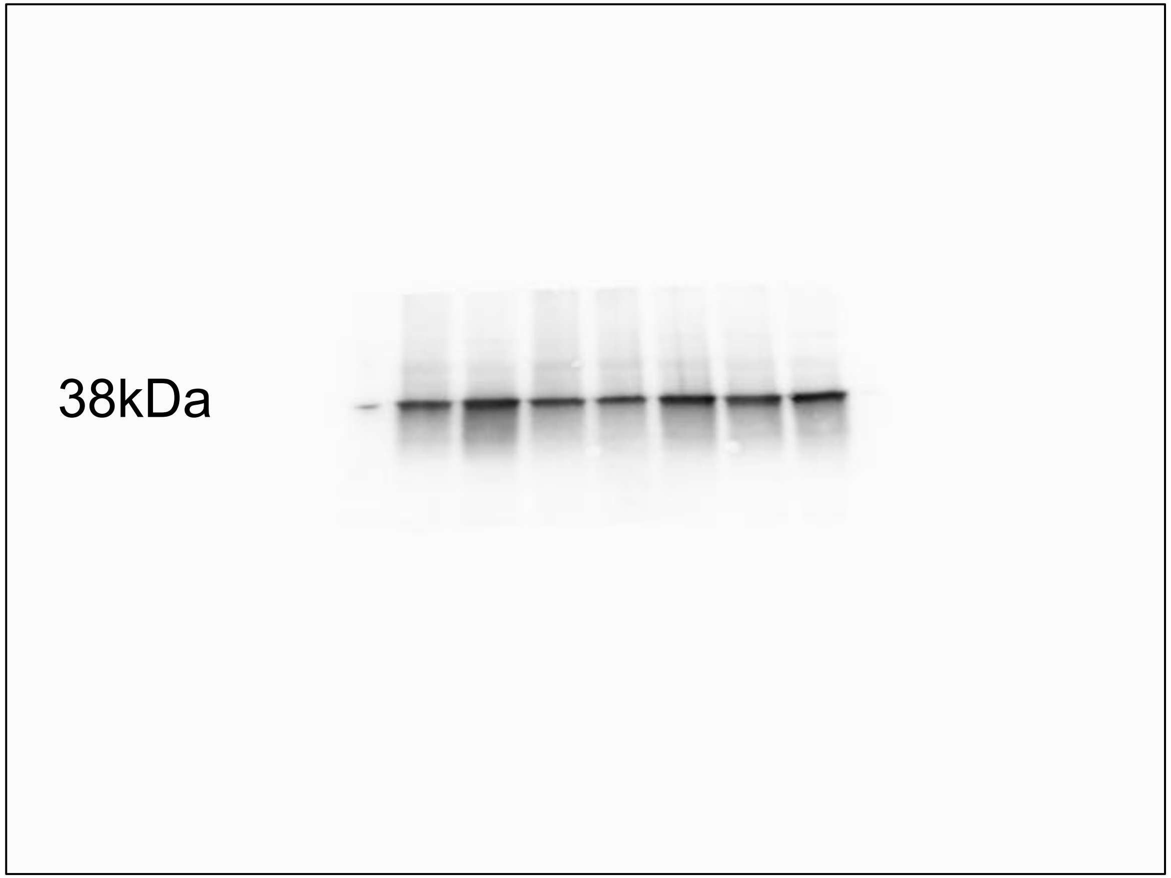

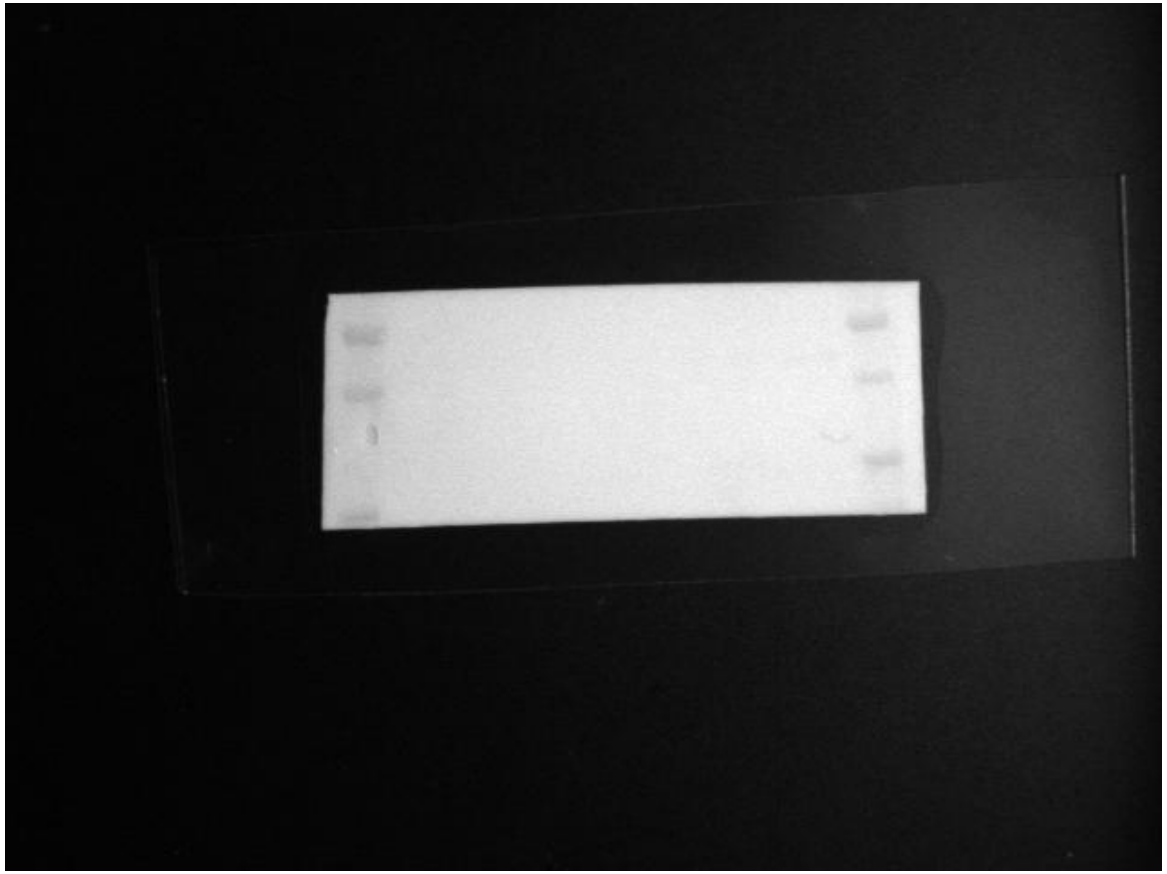

FTD

p-eIF2α

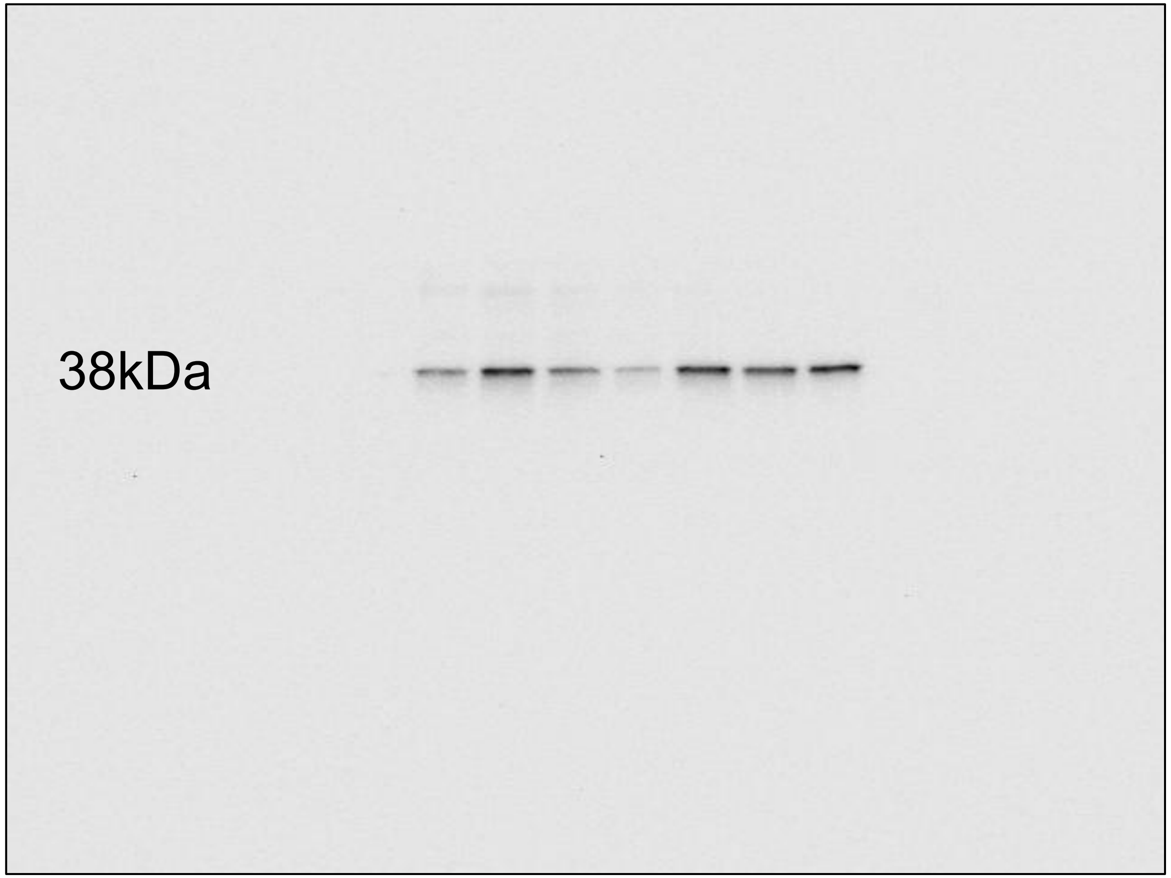

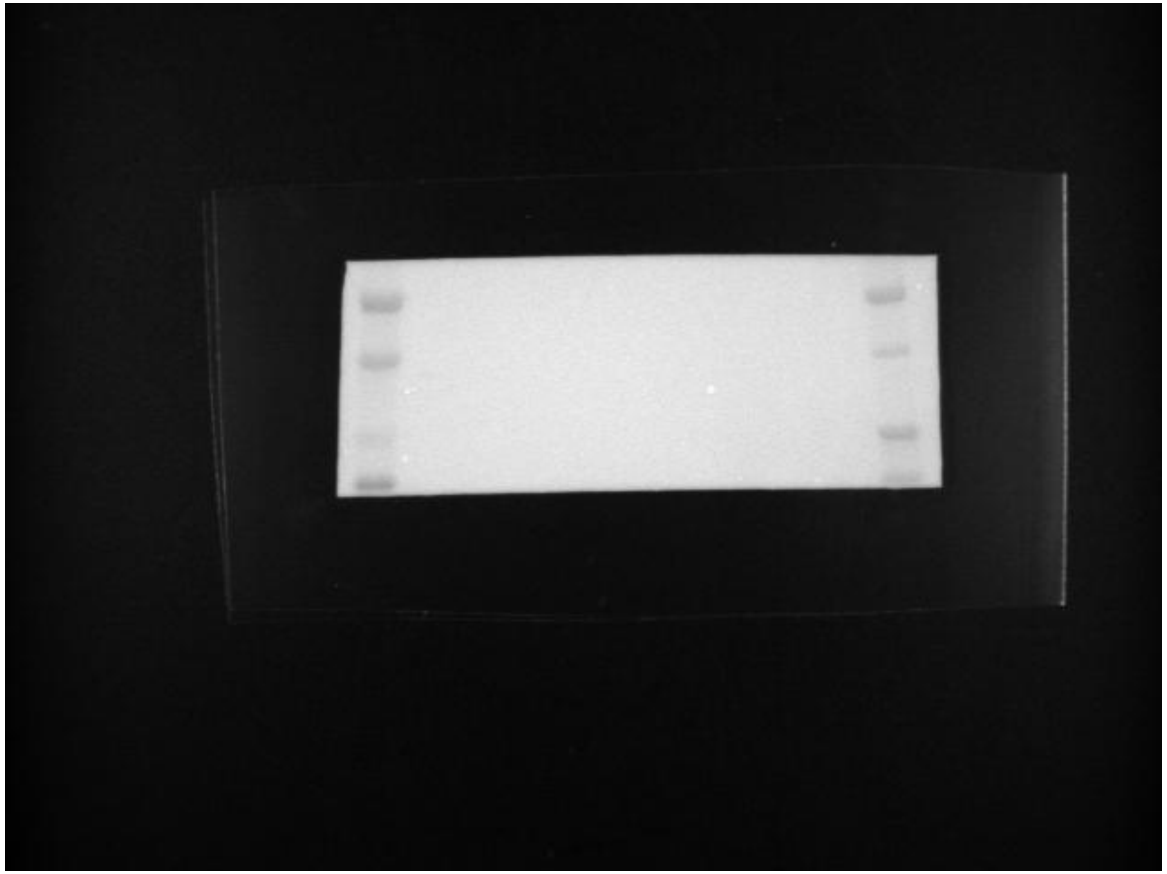

FTD

β-actin

43kDa

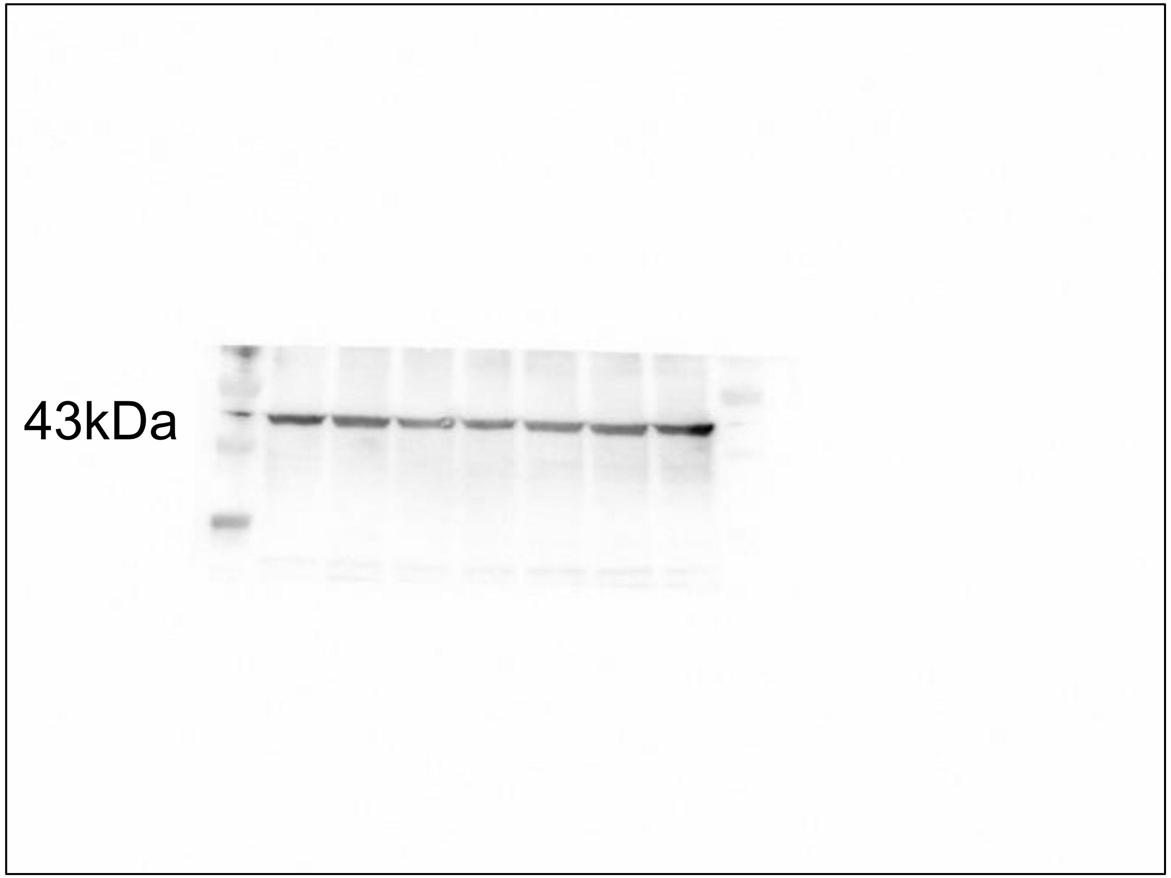

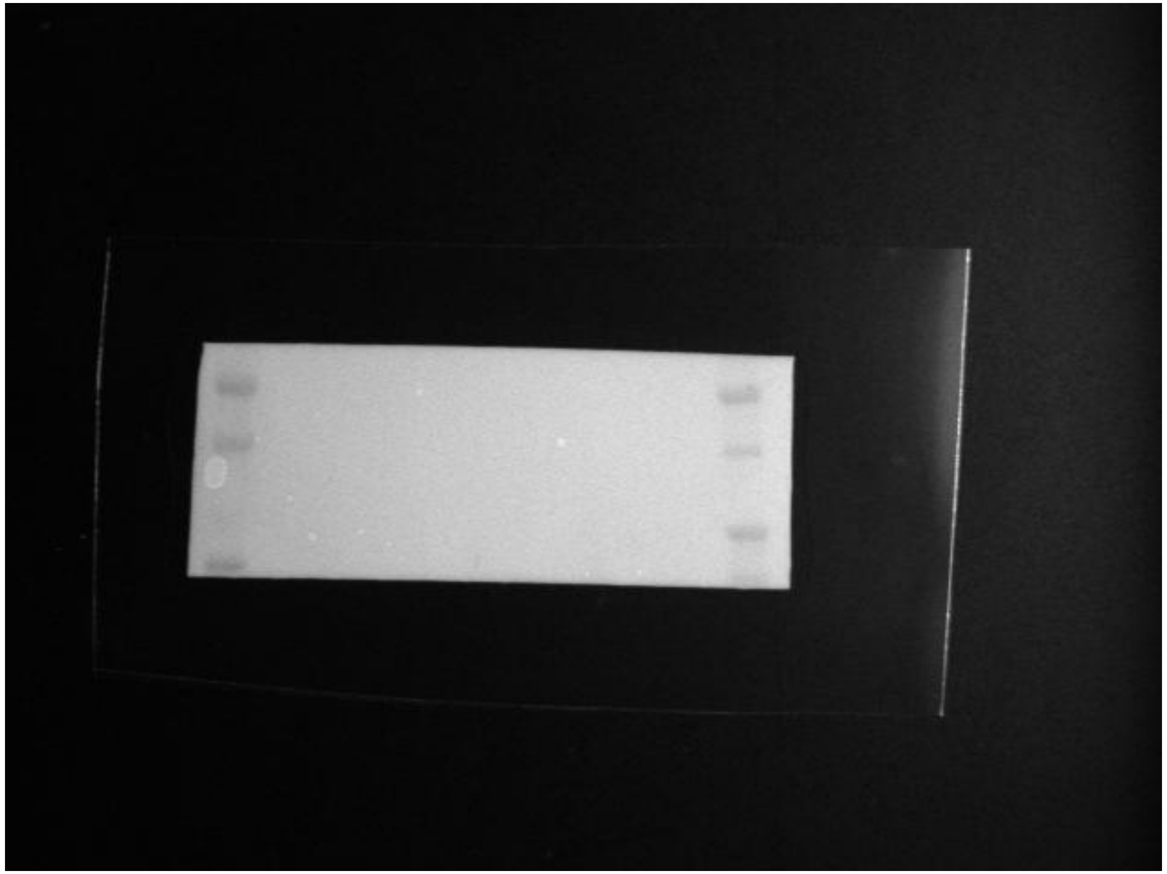

RT

eIF2α

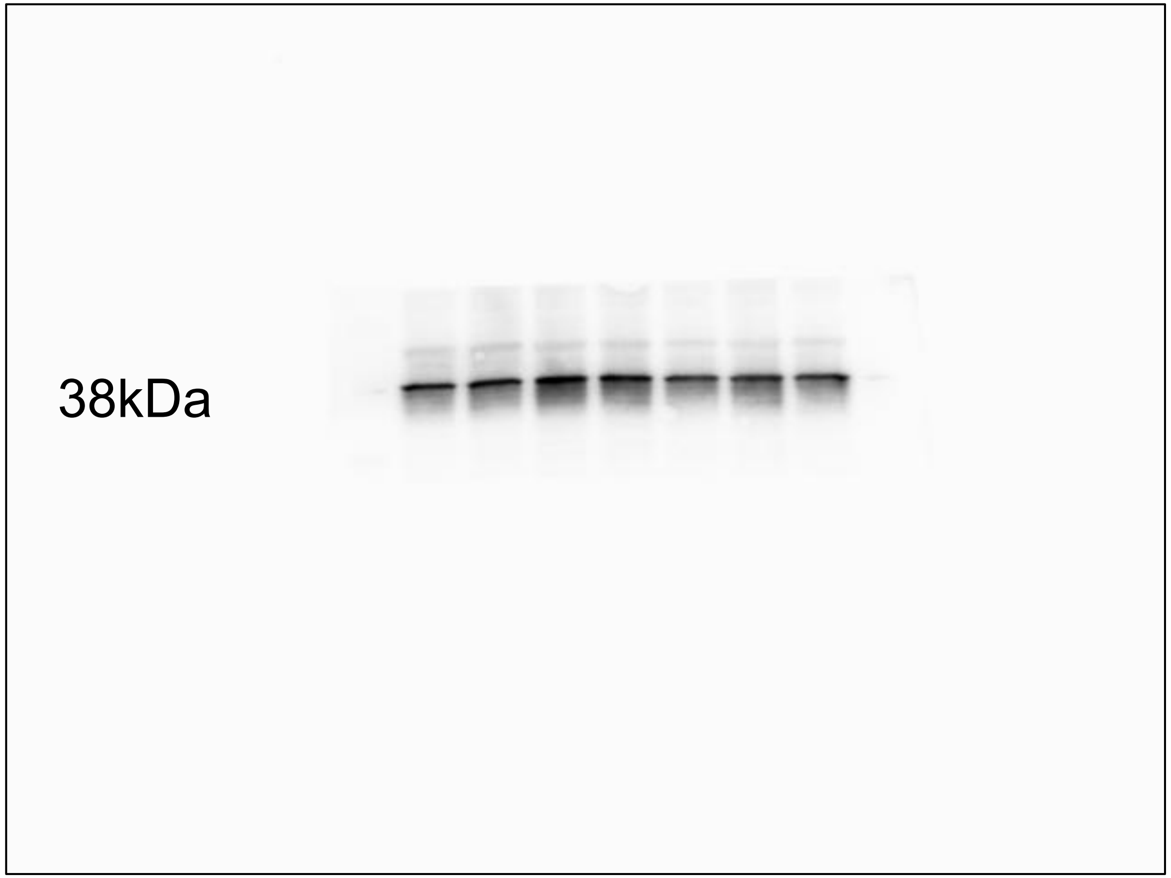

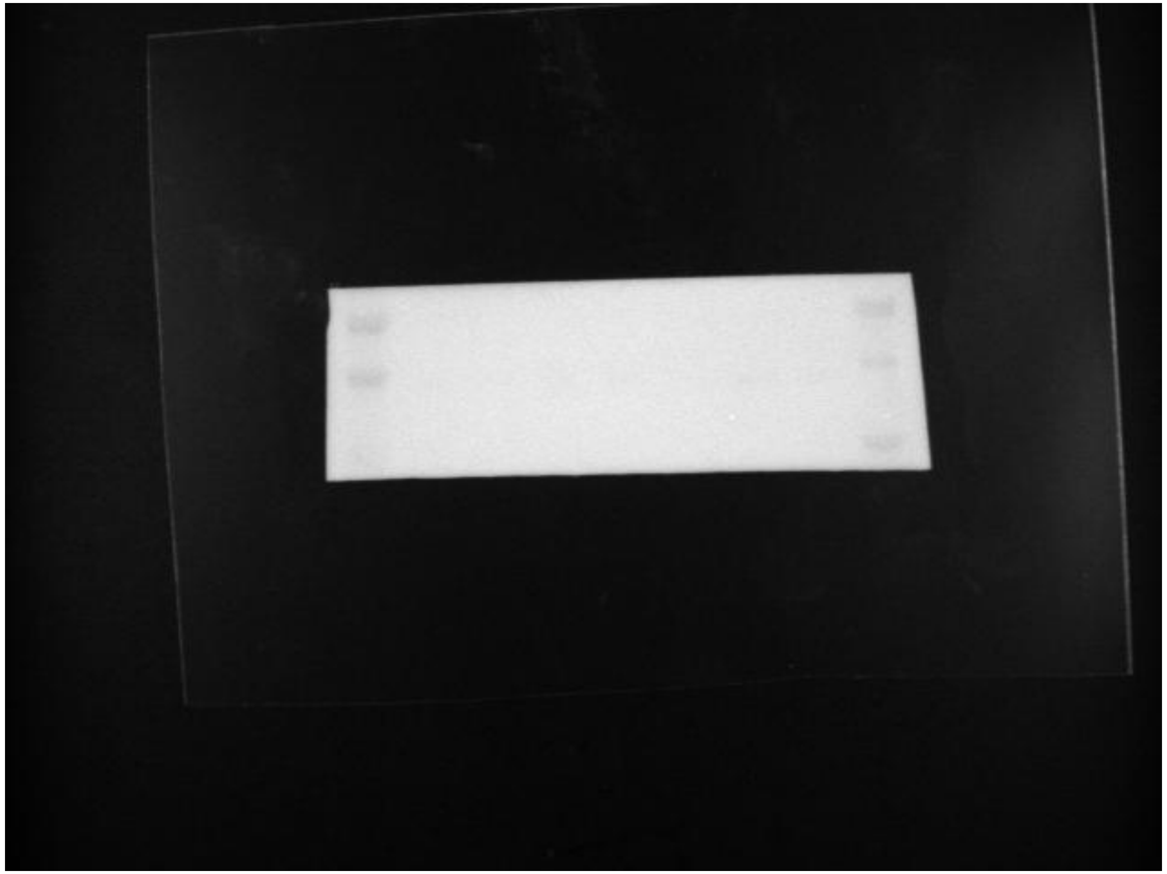

RT

p-eIF2α

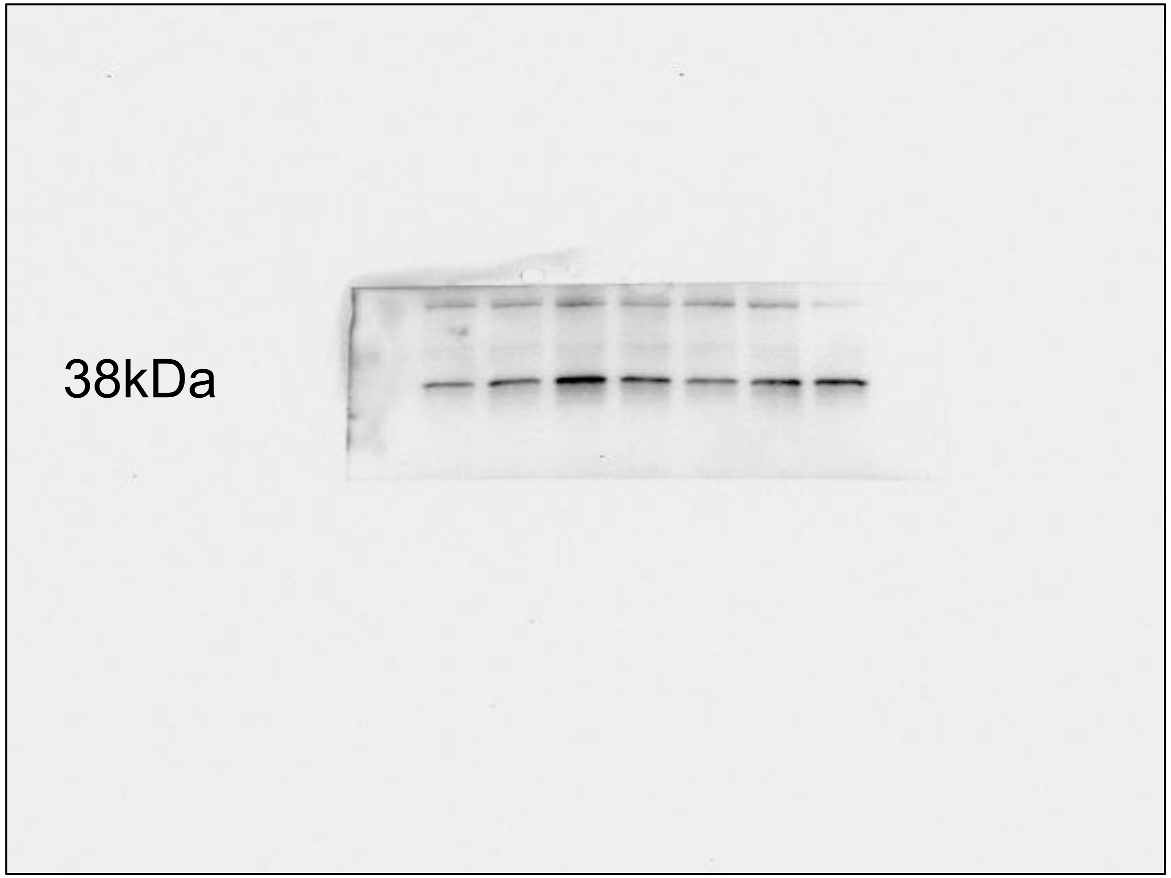

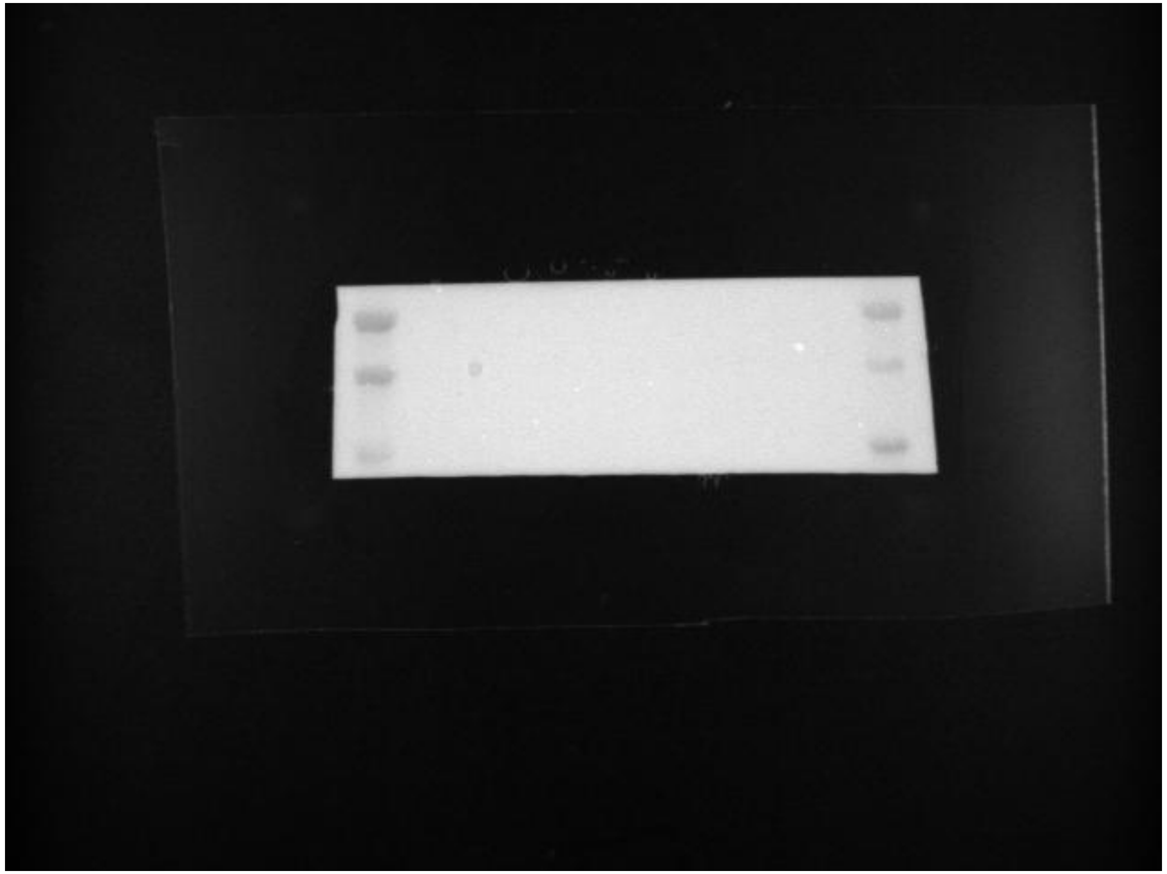

RT

β-actin

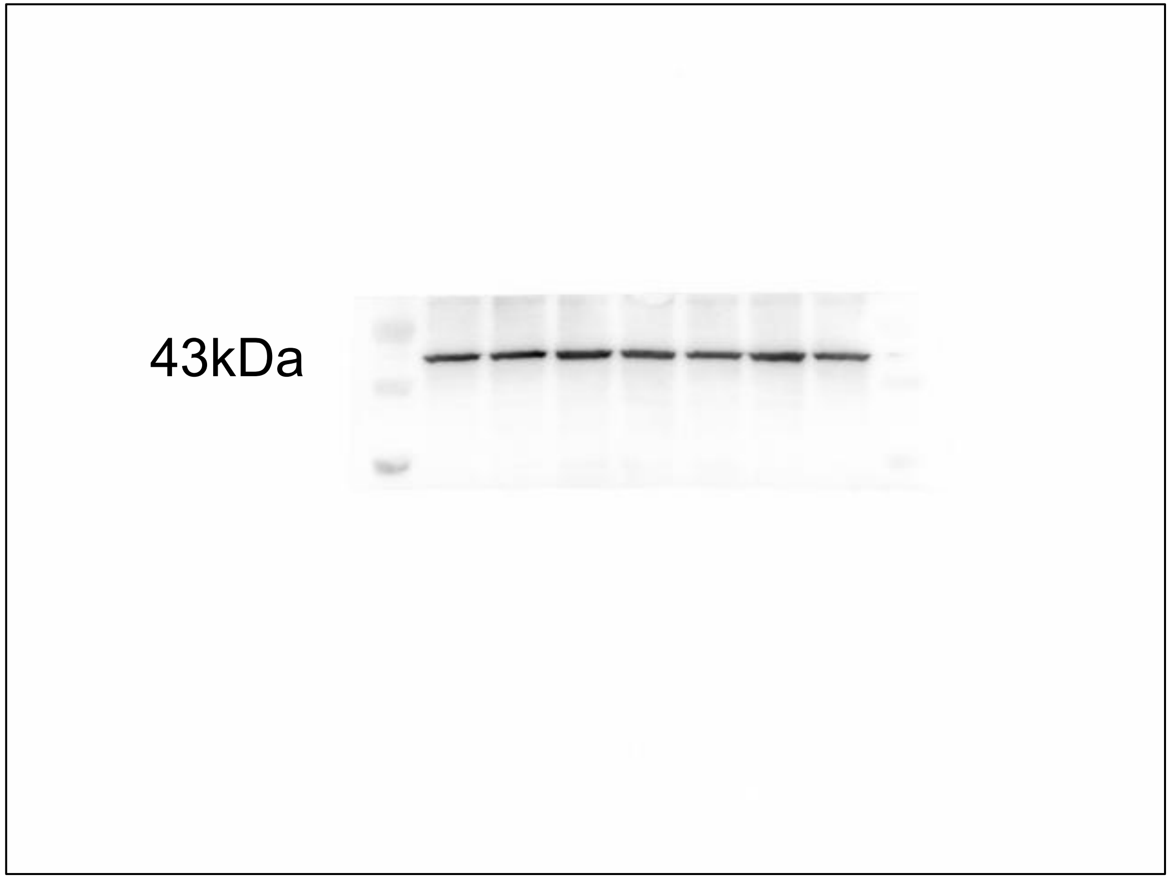

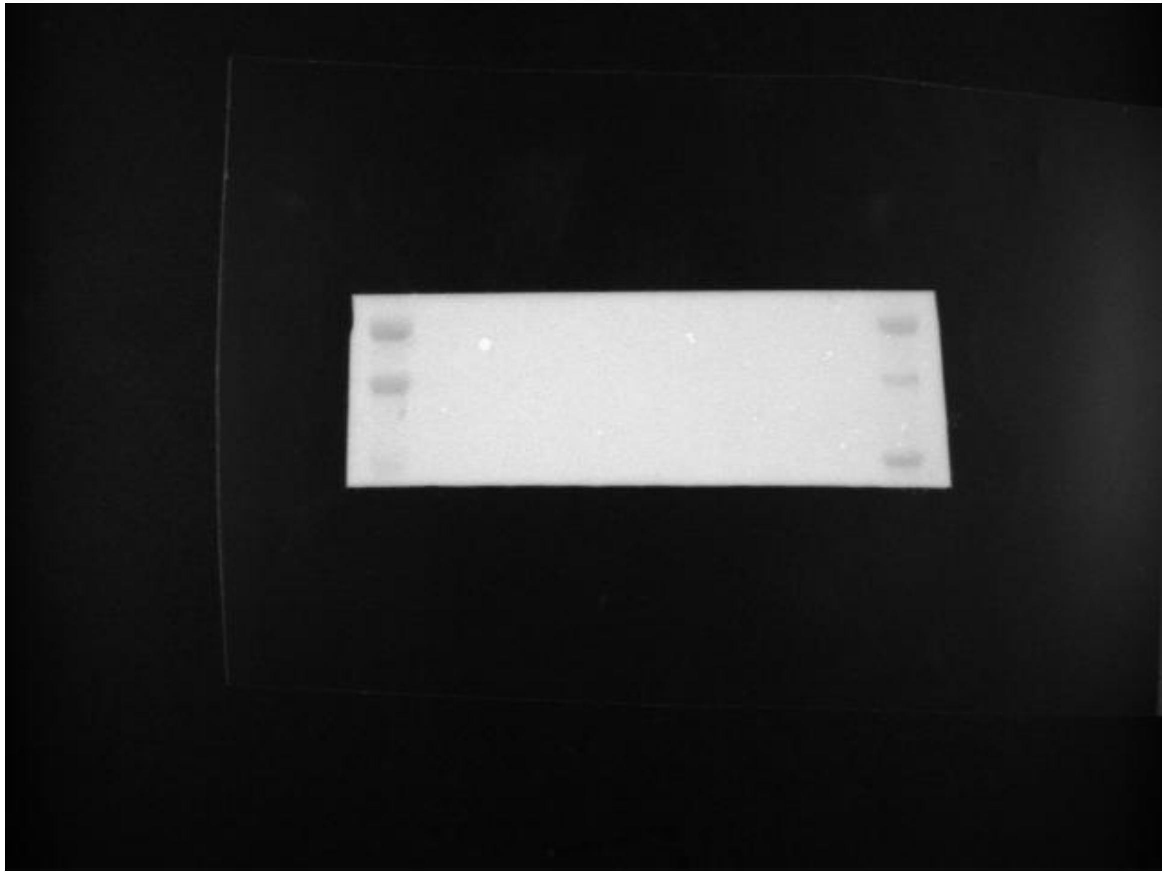

FTD + RT

eIF2α

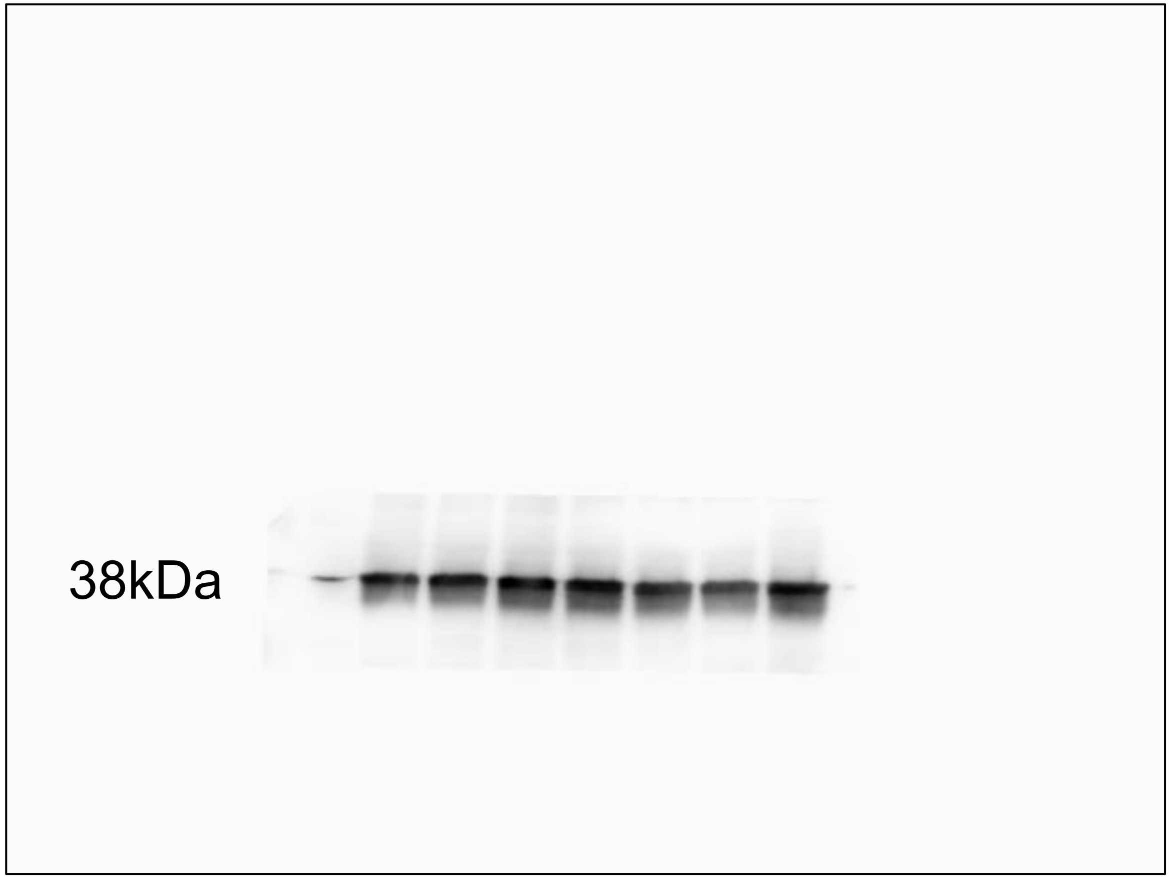

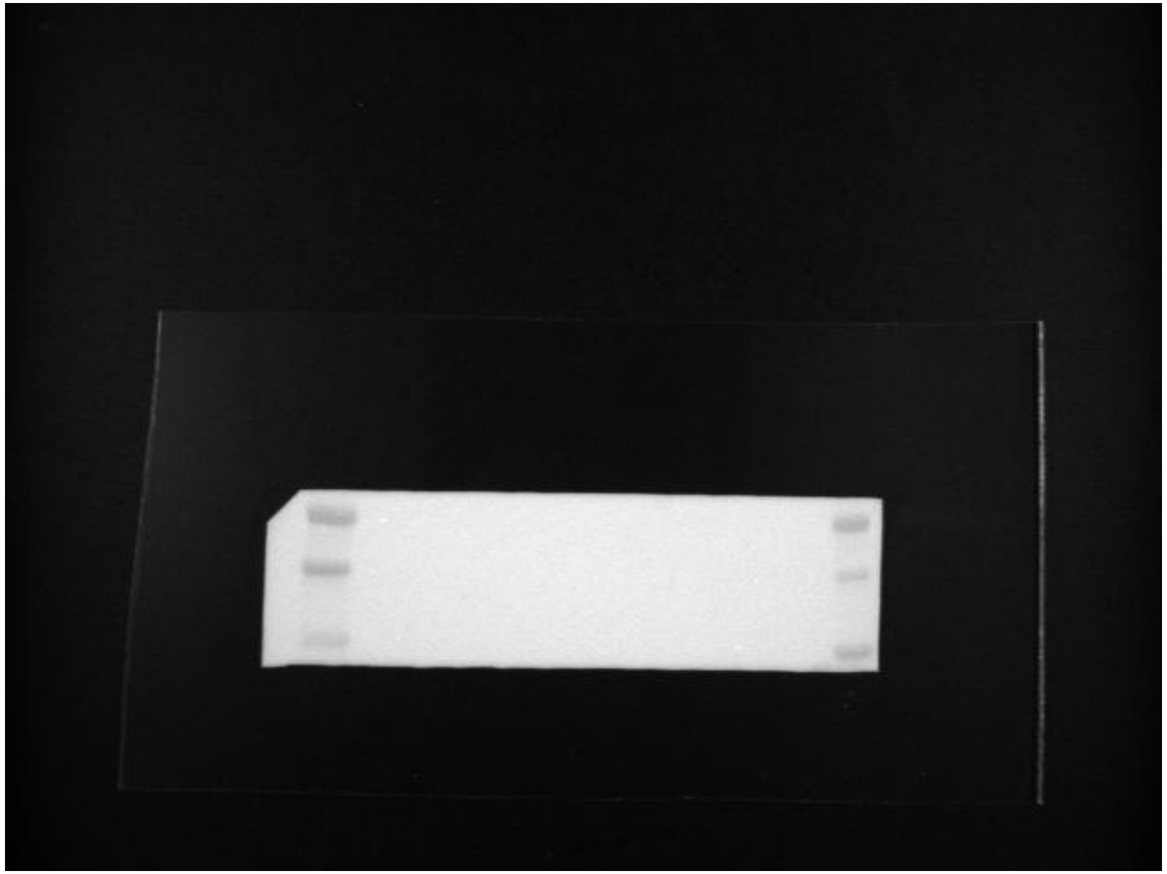

FTD + RT

p-eIF2α

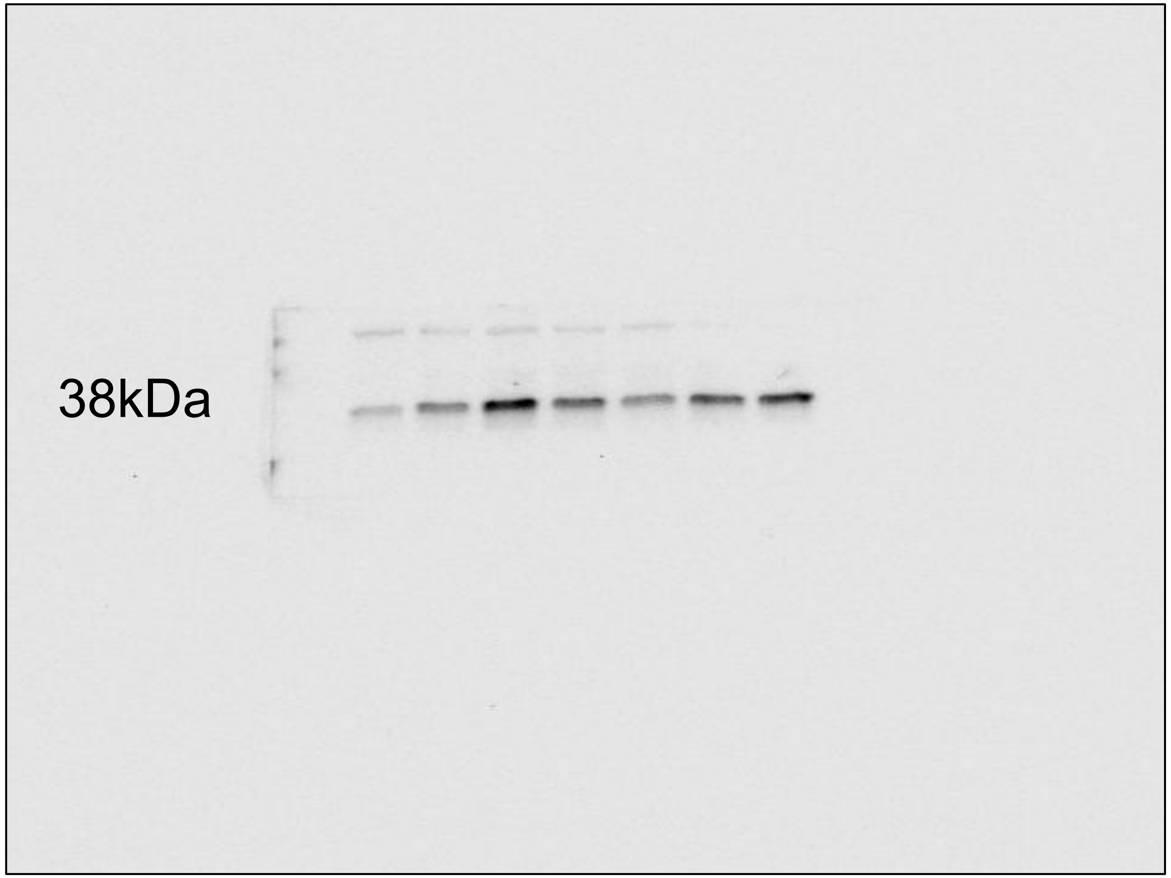

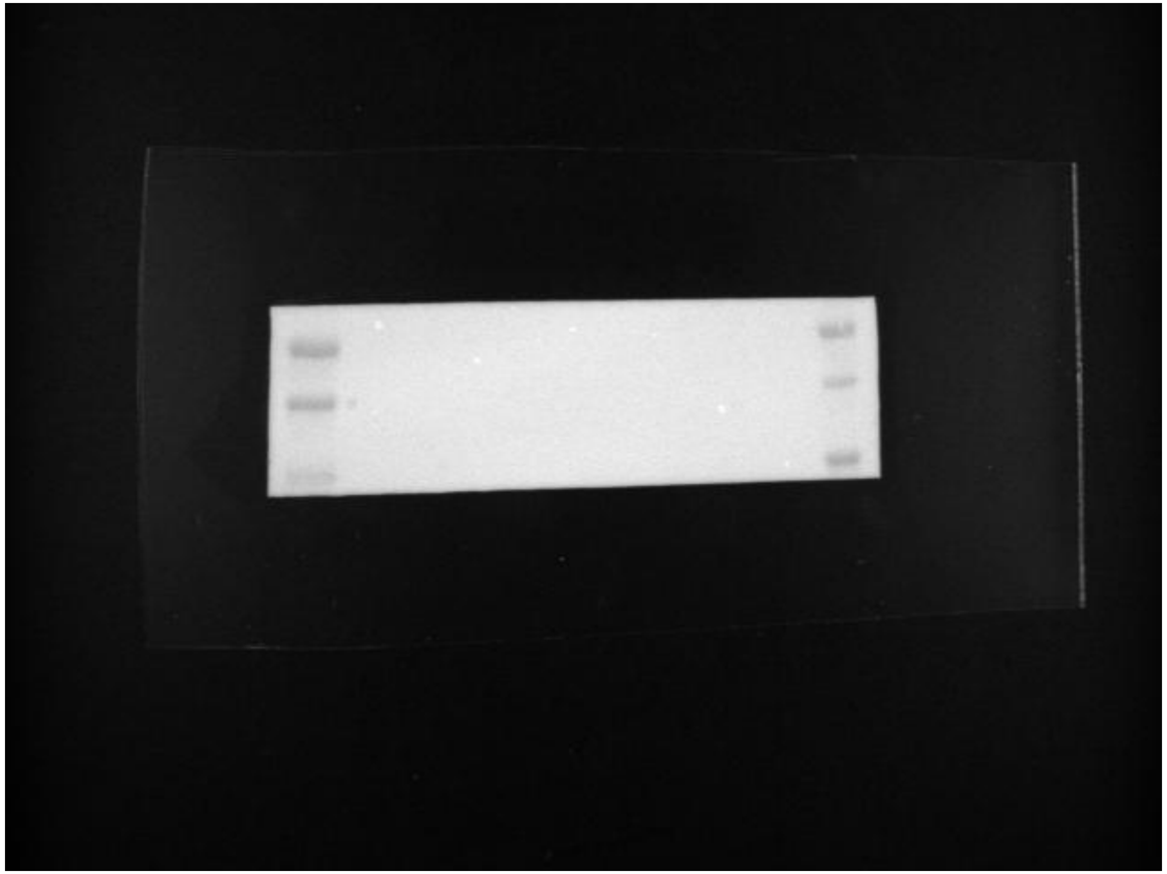

FTD + RT

β-actin

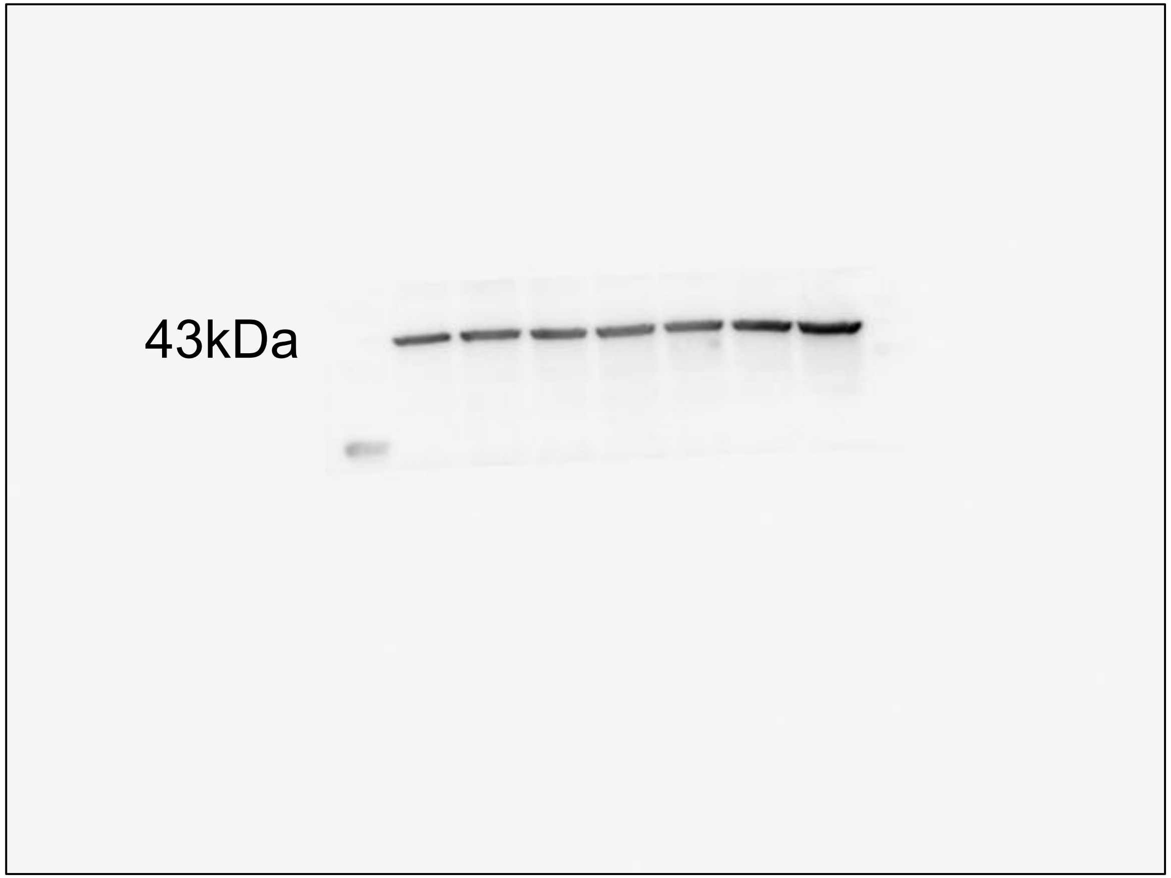

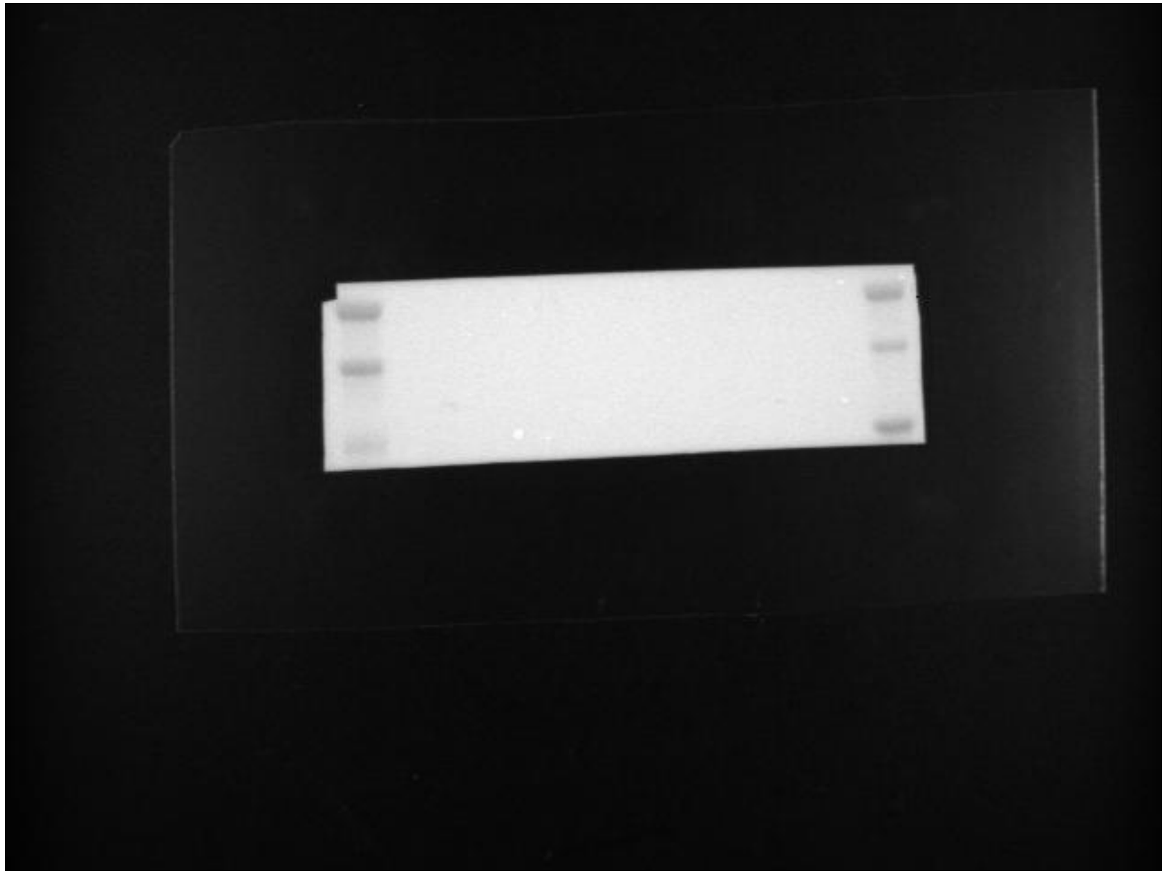

Baseline shortest/longest diameters(mm) and calculated volumes(mm3) of four groups(Control, FTD/TPI, RT, FTD/TPI+RT)

First tumor

	longest diameter	shortest diameter	calclated volumes
Control 1	9.0	8.3	310.0
Control 2	12.1	9.9	593.0
Control 3	11.4	11.4	740.8
Control 4	12.8	9.5	577.6
Control 5	10.7	9.2	452.8
Control 6	11.9	8.5	429.9
FTD/TPI 1	13.3	9.0	538.7
FTD/TPI 2	10.0	9.4	441.8
FTD/TPI 3	13.4	11.0	810.7
FTD/TPI 4	12.0	9.7	564.5
FTD/TPI 5	11.1	10.4	600.3
FTD/TPI 6	11.5	8.1	377.3
RT 1	11.7	9.7	550.4
RT 2	11.7	9.2	495.1
RT 3	13.3	9.6	612.9
RT 4	11.0	8.1	360.9
RT 5	11.1	9.0	449.6
RT 6	11.5	9.1	476.2
FTD/TPI+RT 1	10.9	9.8	523.4
FTD/TPI+RT 2	10.7	8.3	368.6
FTD/TPI+RT 3	10.7	9.6	493.1
FTD/TPI+RT 4	13.3	8.3	458.1
FTD/TPI+RT 5	13.3	11.2	834.2
FTD/TPI+RT 6	10.6	9.7	498.7

	Mean	SEM
Control	517.3	61.7
FTD/TPI	555.5	61.2
RT	490.8	35.2
FTD/TPI+RT	529.3	64.8

Levene's test p=0.75
one-way ANOVA p=0.88

Second tumor

	longest diameter	shortest diameter	calclated volumes
Control 1	6.6	6.0	118.8
Control 2	7.2	5.9	125.3
Control 3	8.8	6.6	191.7
Control 4	7.9	6.3	156.8
Control 5	5.4	5.2	73.0
Control 6	6.8	6.5	143.7
FTD/TPI 1	8.5	6.6	185.1
FTD/TPI 2	8.5	8.2	285.8
FTD/TPI 3	7.2	6.0	129.6
FTD/TPI 4	7.0	6.0	126.0
FTD/TPI 5	6.4	5.7	104.0
FTD/TPI 6	7.1	5.0	88.8
RT 1	7.0	5.7	113.7
RT 2	6.2	5.1	80.6
RT 3	5.9	4.0	47.2
RT 4	5.0	4.7	55.2
RT 5	7.2	5.5	108.9
RT 6	8.2	5.8	137.9
FTD/TPI+RT 1	6.4	5.0	80.0
FTD/TPI+RT 2	7.2	6.1	134.0
FTD/TPI+RT 3	7.5	5.0	93.8
FTD/TPI+RT 4	7.8	5.2	105.5
FTD/TPI+RT 5	7.7	7.3	205.2
FTD/TPI+RT 6	7.5	6.0	135.0

	Mean	SEM
Control	134.9	16.3
FTD/TPI	153.2	29.7
RT	90.6	14.5
FTD/TPI+RT	125.6	18.2

Levene's test p=0.80
one-way ANOVA p=0.21

Supplementary Table S2

Figure 4 Tumor size (Longest diameter (mm), Shortest diameter (mm), Volume (mm3))

First tumor

	day	1	3	6	8	10	13	16
Control 1	Longest	9.0	11.2	12.9	13.8	15.3	18.4	20.0
	Shortest	8.3	10.1	11.0	13.8	15.2	18.1	19.5
	Volume	310.0	571.3	780.5	1314.0	1767.5	3014.0	3802.5
Control 2	Longest	12.1	13.3	15.4	16.8	18.6	19.7	20.0
	Shortest	9.9	10.5	13.3	15.0	15.7	18.3	19.7
	Volume	593.0	733.2	1362.1	1890.0	2292.4	3298.7	3880.9
Control 3	Longest	11.4	12.5	15.0	15.7	17.0	17.3	18.5
	Shortest	11.4	12.0	14.0	15.0	15.7	15.9	17.5
	Volume	740.8	900.0	1470.0	1766.3	2095.2	2186.8	2832.8
Control 4	Longest	12.8	13.9	13.2	14.6	15.3	18.3	19.8
	Shortest	9.5	10.0	12.0	12.5	14.0	16.9	18.0
	Volume	577.6	695.0	950.4	1140.6	1499.4	2613.3	3207.6
Control 5	Longest	10.7	12.5	13.5	15.6	17.4	20.0	20.0
	Shortest	9.2	10.3	11.6	13.3	15.3	17.0	19.5
	Volume	452.8	663.1	908.3	1379.7	2036.6	2890.0	3802.5
Control 6	Longest	11.9	13.0	14.4	15.4	16.3	19.9	20.0
	Shortest	8.5	9.5	11.0	11.8	12.8	16.5	18.4
	Volume	429.9	586.6	871.2	1072.1	1335.3	2708.9	3385.6
FTD/TPI 1	Longest	13.3	15.0	15.6	16.2	16.6	18.5	19.8
	Shortest	9.0	9.8	11.5	12.0	13.2	13.0	14.5
	Volume	538.7	720.3	1031.6	1166.4	1446.2	1563.3	2081.5
FTD/TPI 2	Longest	10.0	11.6	12.2	13.0	14.0	15.7	17.1
	Shortest	9.4	9.6	10.0	10.8	11.3	13.3	15.5
	Volume	441.8	534.5	610.0	758.2	893.8	1388.6	2054.1
FTD/TPI 3	Longest	13.4	14.3	16.0	17.2	19.0	19.5	20.0
	Shortest	11.0	11.5	13.0	13.4	15.0	16.5	18.5
	Volume	810.7	945.6	1352.0	1544.2	2137.5	2654.4	3422.5
FTD/TPI 4	Longest	12.0	13.0	15.2	15.7	17.0	18.7	19.9
	Shortest	9.7	11.0	13.4	14.5	14.9	16.6	18.1
	Volume	564.5	786.5	1364.7	1650.5	1887.1	2576.5	3259.7
FTD/TPI 5	Longest	11.1	13.0	14.9	16.7	16.7	18.0	20.0
	Shortest	10.4	12.0	13.4	15.8	15.8	15.4	15.8
	Volume	600.3	936.0	1337.7	2084.5	2084.5	2134.4	2496.4
FTD/TPI 6	Longest	11.5	12.9	15.0	16.2	16.5	18.9	19.9
	Shortest	8.1	8.7	11.0	11.5	12.3	15.4	17.8
	Volume	377.3	488.2	907.5	1071.2	1248.1	2241.2	3152.6
RT 1	Longest	11.7	12.4	12.0	11.8	12.0	11.8	12.5
	Shortest	9.7	9.5	9.9	9.4	9.4	9.2	9.6
	Volume	550.4	559.6	588.1	521.3	530.2	499.4	576.0
RT 2	Longest	11.7	12.0	11.7	12.8	13.0	12.5	13.1
	Shortest	9.2	9.5	9.3	10.5	10.0	9.5	10.5
	Volume	495.1	541.5	506.0	705.6	650.0	564.1	722.1
RT 3	Longest	13.3	13.4	13.1	13.2	14.3	15.6	16.8
	Shortest	9.6	9.6	10.3	10.4	10.9	11.2	12.1
	Volume	612.9	617.5	694.9	713.9	849.5	978.4	1229.8
RT 4	Longest	11.0	11.0	11.0	11.1	11.9	12.3	13.5

Second tumor

	day	1	3	6	8	10	13	16
Control 1	Longest	7.2	8.1	9.6	10.7	12.0	12.6	13.9
	Shortest	5.9	6.3	8.8	9.4	9.5	10.0	13.0
	Volume	125.3	160.7	371.7	472.7	541.5	630.0	1174.6
Control 2	Longest	8.8	9.5	10.3	11.4	12.6	12.4	13.8
	Shortest	6.6	8.3	9.6	9.8	10.3	10.2	12.1
	Volume	191.7	327.2	474.6	547.4	668.4	645.0	1010.2
Control 3	Longest	6.6	7.5	8.9	9.4	10.9	12.4	14.4
	Shortest	6.0	6.6	8.4	8.7	9.0	10.5	11.4
	Volume	118.8	163.4	314.0	355.7	441.5	683.6	935.7
Control 4	Longest	7.9	8.9	10.0	11.8	13.1	15.6	17.5
	Shortest	6.3	7.9	9.2	9.9	10.5	12.2	14.6
	Volume	156.8	277.7	423.2	578.3	722.1	1161.0	1865.2
Control 5	Longest	5.4	6.4	8.7	10.3	11.5	12.5	14.0
	Shortest	5.2	5.8	6.9	8.3	9.6	10.9	12.1
	Volume	73.0	107.6	207.1	354.8	529.9	742.6	1024.9
Control 6	Longest	6.8	8.5	9.5	10.8	12.8	15.9	18.3
	Shortest	6.5	7.0	8.2	8.8	11.0	12.0	14.2
	Volume	143.7	208.3	319.4	418.2	774.4	1144.8	1845.0
FTD/TPI 1	Longest	8.5	9.1	10.1	11.4	11.4	12.3	13.2
	Shortest	6.6	7.1	7.7	8.5	9.1	10.5	11.9
	Volume	185.1	229.4	299.4	411.8	472.0	678.0	934.6
FTD/TPI 2	Longest	8.5	9.5	10.1	11.5	11.5	12.5	13.5
	Shortest	8.2	8.4	8.7	9.5	9.5	10.3	11.1
	Volume	285.8	335.2	382.2	518.9	518.9	663.1	831.7
FTD/TPI 3	Longest	7.0	8.1	9.2	10.3	10.7	11.9	13.4
	Shortest	6.0	6.3	8.1	9.5	10.2	11.8	13.1
	Volume	126.0	160.7	301.8	464.8	556.6	828.5	1149.8
FTD/TPI 4	Longest	7.1	7.9	8.3	9.5	10.0	10.4	10.8
	Shortest	5.0	5.5	5.9	6.4	6.5	8.6	10.7
	Volume	88.8	119.5	144.5	194.6	211.3	384.6	618.2
FTD/TPI 5	Longest	7.2	8.7	10.0	11.0	12.0	13.4	14.8
	Shortest	6.0	6.8	7.2	8.6	8.9	10.1	11.3
	Volume	129.6	201.1	259.2	406.8	475.3	683.5	944.9
FTD/TPI 6	Longest	6.4	9.2	10.4	11.7	11.9	12.8	13.7
	Shortest	5.7	6.0	9.0	10.1	10.7	10.6	10.8
	Volume	104.0	165.6	421.2	596.8	681.2	719.1	799.0
RT 1	Longest	7.0	7.6	8.3	9.4	10.7	12.4	14.1
	Shortest	5.7	5.8	6.5	6.9	8.0	9.9	10.4
	Volume	113.7	127.8	175.3	223.8	342.4	607.7	762.5
RT 2	Longest	8.2	9.6	10.9	11.9	13.0	14.4	16.3
	Shortest	5.8	6.3	7.3	8.6	9.0	10.4	11.8
	Volume	137.9	190.5	290.4	440.1	526.5	778.8	1134.8
RT 3	Longest	7.2		8.7	9.5	10.8	13.2	14.8
	Shortest	5.5		6.5	7.6	8.3	9.4	10.2
	Volume	108.9		183.8	274.4	372.0	583.2	769.9
RT 4	Longest	5.9	6.7	7.2	7.8	9.5	11.4	12.4

	Shortest	8.1	8.3	9.1	9.7	9.6	10.4	11.0
	Volume	360.9	378.9	455.5	522.2	548.4	665.2	816.8
RT 5	Longest	11.1		11.1	11.8	12.0	13.0	14.1
	Shortest	9.0		9.1	9.1	9.4	9.3	9.8
	Volume	449.6		459.6	488.6	530.2	562.2	677.1
RT 6	Longest	11.5	12.0	12.0	12.4	12.2	12.4	12.9
	Shortest	9.1	9.0	9.6	10.0	9.6	9.0	9.7
	Volume	476.2	486.0	553.0	620.0	562.2	502.2	606.9
FTD/TPI+RT 1	Longest	10.9	11.0	10.9	10.7	10.6	11.2	11.6
	Shortest	9.8	9.1	8.6	8.7	8.3	8.7	9.2
	Volume	523.4	455.5	403.1	404.9	365.1	423.9	490.9
FTD/TPI+RT 2	Longest	10.7	10.2	10.2	10.5	10.6	10.4	10.6
	Shortest	8.3	8.3	8.2	8.6	8.3	9.1	9.8
	Volume	368.6	351.3	342.9	388.3	365.1	430.6	509.0
FTD/TPI+RT 3	Longest	10.7	11.3	11.3	10.3	10.0	10.1	10.2
	Shortest	9.6	8.5	8.6	8.8	8.9	9.2	9.5
	Volume	493.1	408.2	417.9	398.8	396.1	427.4	460.3
FTD/TPI+RT 4	Longest	13.3	12.3	12.4	11.8	11.7	11.4	11.6
	Shortest	8.3	8.6	8.4	8.1	8.4	8.3	8.5
	Volume	458.1	454.9	437.5	387.1	412.8	392.7	419.1
FTD/TPI+RT 5	Longest	13.3	13.3	13.5	12.7	11.2	11.8	12.3
	Shortest	11.2	11.2	10.6	10.5	10.7	11.0	11.6
	Volume	834.2	834.2	758.4	700.1	641.1	713.9	827.5
FTD/TPI+RT 6	Longest	10.6	11.2	10.8	10.5	10.3	10.8	11.5
	Shortest	9.7	10.5	10.3	9.9	10.1	10.8	11.3
	Volume	498.7	617.4	572.9	514.6	525.4	629.9	734.2

	Shortest	4.0	4.7	5.7	6.3	6.5	7.2	8.7
	Volume	47.2	74.0	117.0	154.8	200.7	295.5	469.3
RT 5	Longest	5.0	5.9	6.3	7.0	8.4	9.7	11.0
	Shortest	4.7	5.0	6.2	6.7	7.0	7.4	8.2
	Volume	55.2	73.8	121.1	157.1	205.8	265.6	369.8
RT 6	Longest	6.2	7.2	8.0	9.6	10.5	11.4	13.8
	Shortest	5.1	5.9	6.4	6.9	8.0	9.6	11.4
	Volume	80.6	125.3	163.8	228.5	336.0	525.3	896.7
FTD/TPI+RT 1	Longest	6.4	7.0	6.9	7.2	7.9	9.0	10.1
	Shortest	5.0	5.4	5.4	5.9	6.5	7.7	8.9
	Volume	80.0	102.1	100.6	125.3	166.9	266.8	400.0
FTD/TPI+RT 2	Longest	7.2	7.6	7.7	7.8	8.7	10.0	11.3
	Shortest	6.1	6.6	6.9	6.9	7.3	9.3	10.5
	Volume	134.0	165.5	183.3	185.7	231.8	432.5	622.9
FTD/TPI+RT 3	Longest	7.5	8.3	8.3	8.8	9.4	10.3	11.2
	Shortest	5.0	6.3	6.9	7.0	7.4	8.6	9.8
	Volume	93.8	164.7	197.6	215.6	257.4	380.9	537.8
FTD/TPI+RT 4	Longest	7.8	8.2	7.9	8.2	8.7	9.6	10.5
	Shortest	5.2	5.7	5.7	5.9	6.4	7.0	7.6
	Volume	105.5	133.2	128.3	142.7	178.2	235.2	303.2
FTD/TPI+RT 5	Longest	7.7	8.6	8.7	9.0	9.7	10.5	11.3
	Shortest	7.3	7.7	8.3	8.8	9.7	10.2	10.5
	Volume	205.2	254.9	299.7	348.5	456.3	546.2	622.9
FTD/TPI+RT 6	Longest	7.5	8.2	7.5	7.5	7.7	8.2	9.1
	Shortest	6.0	6.4	6.6	7.0	7.3	7.7	8.1
	Volume	135.0	167.9	163.4	183.8	205.2	243.1	298.5

Baseline shortest/longest diameters(mm) and calculated volumes(mm3) of four groups(Control, FTD/TPI, RT, FTD/TPI+RT)

First tumor

	longest diameter	shortest diameter	calclated volumes
Control 1	10.4	10.0	520.0
Control 2	12.0	11.2	752.6
Control 3	10.7	10.0	535.0
Control 4	11.5	9.1	476.2
Control 5	10.9	9.8	523.4
Control 6	10.0	9.6	460.8
FTD/TPI+RT 1	10.1	10.0	505.0
FTD/TPI+RT 2	9.5	9.3	410.8
FTD/TPI+RT 3	9.4	9.1	389.2
FTD/TPI+RT 4	12.7	10.0	635.0
FTD/TPI+RT 5	11.9	10.8	694.0
FTD/TPI+RT 6	13.0	9.6	599.0
anti PD-1 1	10.3	10.2	535.8
anti PD-1 2	10.7	9.6	493.1
anti PD-1 3	9.4	9.0	380.7
anti PD-1 4	12.0	10.5	661.5
anti PD-1 5	9.4	8.7	355.7
anti PD-1 6	9.3	9.2	393.6
FTD/TPI+RT + anti-PD-1 1	10.0	8.8	387.2
FTD/TPI+RT + anti-PD-1 2	10.8	10.8	629.9
FTD/TPI+RT + anti-PD-1 3	14.4	8.8	557.6
FTD/TPI+RT + anti-PD-1 4	10.5	9.5	473.8
FTD/TPI+RT + anti-PD-1 5	10.0	10.0	500.0
FTD/TPI+RT + anti-PD-1 6	8.9	7.5	250.3

	Mean	SEM
Control	544.7	43.3
FTD/TPI+RT	538.8	50.6
anti-PD-1	470.1	47.8
FTD/TPI+RT + anti-PD-1	466.5	54.5

Levene's test	p=0.73
one-way ANOVA	p=0.54

Second tumor

	longest diameter	shortest diameter	calclated volumes
Control 1	10.8	8.8	418.2
Control 2	10.5	7.5	295.3
Control 3	9.7	9.3	419.5
Control 4	10.0	8.5	361.3
Control 5	8.7	8.5	314.3
Control 6	8.6	8.0	275.2
FTD/TPI+RT 1	10.5	9.4	463.9
FTD/TPI+RT 2	9.9	9.0	401.0
FTD/TPI+RT 3	10.2	9.0	413.1
FTD/TPI+RT 4	9.9	9.5	446.7
FTD/TPI+RT 5	9.9	8.1	324.8
FTD/TPI+RT 6	9.6	8.2	322.8
anti PD-1 1	8.2	8.2	275.7
anti PD-1 2	9.1	8.5	328.7
anti PD-1 3	9.3	8.2	312.7
anti PD-1 4	10.2	9.2	431.7
anti PD-1 5	8.6	8.2	289.1
anti PD-1 6	9.3	7.0	227.9
FTD/TPI+RT + anti-PD-1 1	9.1	8.9	360.4
FTD/TPI+RT + anti-PD-1 2	10.0	8.4	352.8
FTD/TPI+RT + anti-PD-1 3	9.4	8.4	331.6
FTD/TPI+RT + anti-PD-1 4	8.1	8.1	265.7
FTD/TPI+RT + anti-PD-1 5	8.8	8.2	295.9
FTD/TPI+RT + anti-PD-1 6	8.9	8.1	292.0

	Mean	SEM
Control	347.3	25.4
FTD/TPI+RT	395.4	24.5
anti-PD-1	311.0	28.0
FTD/TPI+RT + anti-PD-1	316.4	15.4

Levene's test	p=0.74
one-way ANOVA	p=0.08

Supplementary Table S4

Figure 6 Tumor size (Longest diameter (mm), Shortest diameter (mm), Volume (mm3))

First tumor

	day	1	3	6	8	10	13	16
Control 1	Longest	10.4	11.2	13.0	16.0	16.5	16.6	17.5
	Shortest	10.0	10.7	13.0	15.0	16.0	16.0	17.0
	Volume	520.0	641.1	1098.5	1800.0	2112.0	2124.8	2528.8
Control 2	Longest	12.0	13.7	14.8	17.9	18.5	19.0	20.0
	Shortest	11.2	12.6	13.8	15.5	17.3	18.0	19.0
	Volume	752.6	1087.5	1409.3	2150.2	2768.4	3078.0	3610.0
Control 3	Longest	10.7	13.0	14.5	15.7	17.8	19.0	20.0
	Shortest	10.0	11.6	13.5	14.7	17.6	18.7	19.8
	Volume	535.0	874.6	1321.3	1696.3	2756.9	3322.1	3920.4
Control 4	Longest	11.5	13.6	14.8	16.5	17.6	18.8	19.5
	Shortest	9.1	11.0	12.0	15.4	16.6	17.7	19.0
	Volume	476.2	822.8	1065.6	1956.6	2424.9	2944.9	3519.8
Control 5	Longest	10.9	12.0	14.0	15.9	16.5	17.0	17.6
	Shortest	9.8	11.7	13.2	15.2	16.2	16.5	17.2
	Volume	523.4	821.3	1219.7	1836.8	2165.1	2314.1	2603.4
Control 6	Longest	10.0	12.0	13.6	15.1	17.4	18.6	19.5
	Shortest	9.6	10.6	12.7	14.5	15.0	18.0	19.0
	Volume	460.8	674.2	1096.8	1587.4	1957.5	3013.2	3519.8
FTD/TPI+RT 1	Longest	10.1	10.1	11.4	11.4	10.5	10.7	11.5
	Shortest	10.0	10.0	10.0	10.8	10.5	10.4	11.2
	Volume	505.0	505.0	570.0	664.8	578.8	578.7	721.3
FTD/TPI+RT 2	Longest	9.5	10.3	11.0	12.0	12.5	12.0	13.2
	Shortest	9.3	9.8	10.4	11.0	11.0	11.0	11.0
	Volume	410.8	494.6	594.9	726.0	756.3	726.0	798.6
FTD/TPI+RT 3	Longest	9.4	9.7	10.5	10.5	11.0	12.0	12.7
	Shortest	9.1	9.4	10.0	10.4	11.0	11.0	11.0
	Volume	389.2	428.5	525.0	567.8	665.5	726.0	768.4
FTD/TPI+RT 4	Longest	12.7	13.3	13.7	14.0	14.8	15.3	16.2
	Shortest	10.0	10.6	10.7	11.1	11.6	12.3	12.0
	Volume	635.0	747.2	784.3	862.5	995.7	1157.4	1166.4
FTD/TPI+RT 5	Longest	11.9	13.0	13.2	13.5	14.0	15.5	16.2
	Shortest	10.8	10.5	11.2	10.9	11.0	11.0	11.3
	Volume	694.0	716.6	827.9	802.0	847.0	937.8	1034.3
FTD/TPI+RT 6	Longest	13.0	13.4	14.0	14.8	15.0	15.5	16.7
	Shortest	9.6	10.5	11.9	11.4	12.0	12.2	12.8
	Volume	599.0	738.7	991.3	961.7	1080.0	1153.5	1368.1
anti-PD-1 1	Longest	10.3	11.5	13.4	15.0	16.3	17.0	19.0
	Shortest	10.2	11.3	13.2	14.5	14.6	17.0	19.0
	Volume	535.8	734.2	1167.4	1576.9	1737.3	2456.5	3429.5
anti-PD-1 2	Longest	10.7	11.4	12.0	14.5	16.0	17.5	18.5
	Shortest	9.6	11.0	12.0	13.6	14.3	14.9	15.4
	Volume	493.1	689.7	864.0	1341.0	1635.9	1942.6	2193.7
anti-PD-1 3	Longest	9.4	10.7	11.8	13.2	14.1	14.2	15.0
	Shortest	9.0	10.6	11.8	13.0	13.6	14.2	14.2
	Volume	380.7	601.1	821.5	1115.4	1304.0	1431.6	1512.3
anti-PD-1 4	Longest	12.0	13.3	14.0	15.4	16.5	17.0	17.6
	Shortest	10.5	11.4	12.0	13.0	14.5	16.0	16.0
	Volume	661.5	864.2	1008.0	1301.3	1734.6	2176.0	2252.8
anti-PD-1 5	Longest	9.4	10.9	12.0	13.9	15.3	17.7	19.0
	Shortest	8.7	9.2	11.6	13.1	15.1	16.0	18.0
	Volume	355.7	461.3	807.4	1192.7	1744.3	2265.6	3078.0
anti-PD-1 6	Longest	9.3	10.8	12.7	13.6	14.9	16.6	17.0
	Shortest	9.2	10.5	12.1	13.0	14.2	16.0	16.0
	Volume	393.6	595.4	929.7	1149.2	1502.2	2124.8	2176.0
FTD/TPI+RT + anti-PD-1 1	Longest	10.0	10.8	9.3	7.8	8.0	8.0	8.9
	Shortest	8.8	8.7	8.0	7.8	7.7	7.4	8.7
	Volume	387.2	408.7	297.6	237.3	237.2	219.0	336.8
FTD/TPI+RT + anti-PD-1 2	Longest	10.8	11.8	11.4	12.1	12.5	12.5	12.7
	Shortest	10.8	10.7	10.4	11.5	11.2	11.4	11.7
	Volume	629.9	675.5	616.5	800.1	784.0	812.3	869.3
FTD/TPI+RT + anti-PD-1 3	Longest	14.4	15.6	15.9	16.5	17.4	18.4	19.0
	Shortest	8.8	9.3	10.1	10.0	10.5	10.5	10.7

Second tumor

	day	1	3	6	8	10	13	16
Control 1	Longest	10.8	11.7	14.0	15.1	16.4	16.0	17.5
	Shortest	8.8	9.7	10.5	11.7	12.5	11.5	13.5
	Volume	418.2	550.4	771.8	1033.5	1281.3	1058.0	1594.7
Control 2	Longest	10.5	11.4	13.0	14.8	16.0	17.5	18.8
	Shortest	7.5	9.0	10.0	11.3	12.6	13.7	14.4
	Volume	295.3	461.7	650.0	944.9	1270.1	1642.3	1949.2
Control 3	Longest	9.7	11.0	12.8	14.7	16.0	17.0	19.6
	Shortest	9.3	10.3	12.7	13.0	14.0	15.0	16.0
	Volume	419.5	583.5	1032.3	1242.2	1568.0	1912.5	2508.8
Control 4	Longest	10.0	11.7	14.7	15.5	17.3	18.3	19.0
	Shortest	8.5	10.0	10.7	13.2	14.0	15.0	15.7
	Volume	361.3	585.0	841.5	1350.4	1695.4	2058.8	2341.7
Control 5	Longest	8.7	10.5	11.9	13.0	14.3	15.0	16.0
	Shortest	8.5	8.5	9.9	11.5	13.0	13.6	13.6
	Volume	314.3	379.3	583.2	859.6	1208.4	1387.2	1479.7
Control 6	Longest	8.6	10.6	11.6	13.0	14.7	15.9	16.5
	Shortest	8.0	9.7	10.9	12.7	13.6	14.9	15.0
	Volume	275.2	498.7	689.1	1048.4	1359.5	1765.0	1856.3
FTD/TPI+RT 1	Longest	10.5	10.5	11.4	13.5	13.5	14.0	15.5
	Shortest	9.4	9.6	11.0	13.2	12.5	13.2	14.5
	Volume	463.9	483.8	689.7	1176.1	1054.7	1219.7	1629.4
FTD/TPI+RT 2	Longest	9.9	10.0	10.9	12.2	13.7	14.6	15.9
	Shortest	9.0	9.6	9.5	10.3	11.6	12.2	14.8
	Volume	401.0	460.8	491.9	647.1	921.7	1086.5	1741.4
FTD/TPI+RT 3	Longest	10.2	10.5	11.1	12.2	13.0	14.2	15.0
	Shortest	9.0	9.3	10.5	12.2	12.5	13.0	14.3
	Volume	413.1	454.1	611.9	907.9	1015.6	1199.9	1533.7
FTD/TPI+RT 4	Longest	9.9	10.0	11.1	12.1	12.7	13.4	15.2
	Shortest	9.5	9.5	10.5	11.2	12.1	12.8	13.3
	Volume	446.7	451.7	611.9	758.9	929.7	1097.7	1344.4
FTD/TPI+RT 5	Longest	9.9	9.9	11.0	12.4	13.0	15.0	16.1
	Shortest	8.1	8.9	9.4	10.5	11.6	13.0	14.0
	Volume	324.8	392.1	486.0	683.6	874.6	1267.5	1577.8
FTD/TPI+RT 6	Longest	9.6	10.0	11.3	12.4	13.2	14.4	17.0
	Shortest	8.2	8.3	9.4	10.1	11.4	12.5	15.0
	Volume	322.8	344.5	499.2	632.5	857.7	1125.0	1912.5
anti-PD-1 1	Longest	8.2	9.2	11.6	12.6	13.2	14.4	15.5
	Shortest	8.2	8.9	10.0	11.0	12.0	14.0	15.2
	Volume	275.7	364.4	580.0	762.3	950.4	1411.2	1790.6
anti-PD-1 2	Longest	9.1	10.0	11.3	13.6	14.0	14.3	15.0
	Shortest	8.5	9.2	9.5	12.2	12.5	13.6	14.0
	Volume	328.7	423.2	509.9	1012.1	1093.8	1322.5	1470.0
anti-PD-1 3	Longest	9.3	10.5	11.6	13.0	13.4	14.0	14.0
	Shortest	8.2	9.2	9.5	10.3	10.5	10.5	10.5
	Volume	312.7	444.4	523.5	689.6	738.7	771.8	771.8
anti-PD-1 4	Longest	10.2	10.5	12.0	12.5	13.1	13.6	14.4
	Shortest	9.2	10.2	12.0	12.3	12.7	13.0	13.4
	Volume	431.7	546.2	864.0	945.6	1056.4	1149.2	1292.8
anti-PD-1 5	Longest	8.6	9.8	10.7	11.8	13.6	14.6	16.6
	Shortest	8.2	9.2	10.6	11.3	13.0	14.0	15.6
	Volume	289.1	414.7	601.1	753.4	1149.2	1430.8	2019.9
anti-PD-1 6	Longest	9.3	10.7	11.3	12.5	13.0	14.6	14.2
	Shortest	7.0	8.2	10.0	11.1	11.0	11.5	11.0
	Volume	227.9	359.7	565.0	770.1	786.5	965.4	859.1
FTD/TPI+RT + anti-PD-1 1	Longest	9.1	9.0	9.1	8.9	9.0	9.7	9.8
	Shortest	8.9	8.5	8.4	7.7	8.0	7.7	8.1
	Volume	360.4	325.1	321.0	263.8	288.0	287.6	321.5
FTD/TPI+RT + anti-PD-1 2	Longest	10.0	10.2	10.7	12.0	12.5	13.3	14.0
	Shortest	8.4	8.3	9.2	10.0	11.0	11.5	12.6
	Volume	352.8	351.3	452.8	600.0	756.3	879.5	1111.3
FTD/TPI+RT + anti-PD-1 3	Longest	9.4	9.3	9.7	11.2	11.2	11.6	12.0
	Shortest	8.4	8.7	8.8	9.5	10.3	11.4	11.8

Supplementary Table S4

	Volume	557.6	674.6	811.0	825.0	959.2	1014.3	1087.7
FTD/TPI+RT + anti-PD-1 4	Longest	10.5	10.7	11.0	11.4	11.7	12.3	12.7
	Shortest	9.5	10.5	10.9	11.3	11.5	11.5	11.5
	Volume	473.8	589.8	653.5	727.8	773.7	813.3	839.8
FTD/TPI+RT + anti-PD-1 5	Longest	10.0	10.6	10.5	10.6	10.9	12.0	12.2
	Shortest	10.0	10.5	10.0	10.2	10.4	10.9	11.5
	Volume	500.0	584.3	525.0	551.4	589.5	712.9	806.7
FTD/TPI+RT + anti-PD-1 6	Longest	8.9	9.3	9.0	8.3	9.2	9.4	9.8
	Shortest	7.5	7.4	7.5	7.6	7.4	7.9	8.2
	Volume	250.3	254.6	253.1	239.7	251.9	293.3	329.5

	Volume	331.6	352.0	375.6	505.4	594.1	753.8	835.4
FTD/TPI+RT + anti-PD-1 4	Longest	8.1	8.6	9.4	11.6	12.5	13.6	14.0
	Shortest	8.1	8.3	8.5	9.7	10.1	11.0	11.5
	Volume	265.7	296.2	339.6	545.7	637.6	822.8	925.8
FTD/TPI+RT + anti-PD-1 5	Longest	8.8	9.7	9.2	10.9	11.2	12.0	12.5
	Shortest	8.2	8.4	9.2	10.5	10.9	11.2	11.5
	Volume	295.9	342.2	389.3	600.9	665.3	752.6	826.6
FTD/TPI+RT + anti-PD-1 6	Longest	8.9	8.4	8.7	9.0	10.0	10.8	11.6
	Shortest	8.1	8.1	8.4	8.8	8.8	9.0	9.7
	Volume	292.0	275.6	306.9	348.5	387.2	437.4	545.7
